# Exploration of novel 6,8,9-trisubstituted purine analogues: synthesis, in vitro biological evaluation, and their effect on human cancer cells

**DOI:** 10.55730/1300-0527.3643

**Published:** 2023-12-04

**Authors:** Muhammed Fatih POLAT, İrem DURMAZ ŞAHİN, Rengül ATALAY, Meral TUNÇBİLEK

**Affiliations:** 1 Department of Pharmaceutical Basic Sciences, Faculty of Pharmacy, Erzincan Binali Yıldırım University, Erzincan, Turkiye; 2 Research Center for Translational Medicine (KUTTAM), Koç University, İstanbul, Turkiye; 3 Department of Medical Biology, School of Medicine, Koç University, İstanbul, Turkiye; 4 Cancer Systems Biology Laboratory, Graduate School of Informatics, ODTU, Ankara, Turkiye; 5 Section of Pulmonary and Critical Care Medicine, University of Chicago, Chicago, USA; 6 Department of Pharmaceutical Chemistry, Faculty of Pharmacy, Ankara University, Ankara, Turkiye

**Keywords:** 6, 8, 9-Trisubstituted purine analogs, synthesis, cytotoxic activity, human epithelial cancer cells

## Abstract

Cancer, a leading global cause of mortality, demands continuous advancements in therapeutic strategies. This study focuses on the design and synthesis of a novel series of purine derivatives, specifically 6-(substituted phenyl piperazine)-8-(4-phenoxyphenyl)-9-cyclopentyl purine derivatives (5–11). The motivation behind this endeavor lies in addressing acquired resistance mechanisms in cancer cells, a significant hurdle in current treatment modalities. The synthesis, starting from 4,6-dichloro-5-nitropyrimidine, involves a multi-step process, resulting in seven new purine derivatives.

Biological evaluation against human liver, colon, and breast cancer cells (Huh7, HCT116, and MCF7, respectively) was performed using the SRB assay. Among the synthesized analogs, compounds 5 and 6, exhibited notable cytotoxic activity, surpassing clinically used positive controls 5-Fluorouracil and Fludarabine in terms of efficacy. This research underscores the potential of purine derivatives with a phenyl group at the C-8 position as a scaffold for developing compounds with improved anticancer properties. The findings offer insights for future exploration and development of novel agents in cancer pharmaceutical research.

## 1. Introduction

Cancer, a pervasive cause of death on a global scale, accounted for approximately 10 million fatalities in the year 2020, as reported by GLOBOCAN statistics [1]. Existing treatment options encompass chemotherapy, hormone therapy, immunotherapy, and γ-radiation. However, the emergence of acquired resistance mechanisms in cancer cells poses a significant obstacle to effective disease management, compounded by the adverse effects of the drugs employed. Consequently, the exploration of novel compounds with anticancer properties becomes imperative for advancing cancer pharmaceutical research.

**Figure 1:**
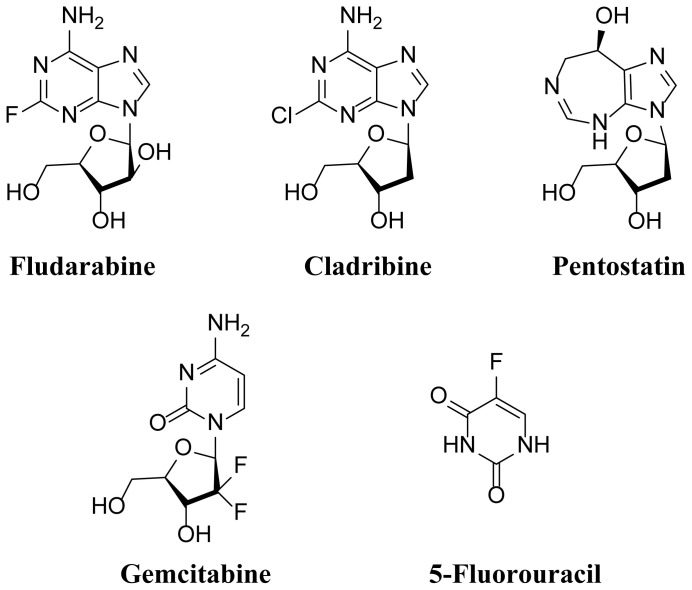
Structures of Fludarabine, Cladribine, Pentostatin, Gemcitabine, and 5-Fluorouracil.

Analogues of purine nucleosides, and purine/pyrimidine nucleobase have found utility in the therapeutics of cancer. Purine analogues play vital roles in many cellular bioprocesses including but not limited to cell growth, proliferation, and division [2]. Consequently, these molecules assume substantial importance in cancer therapy. These analogues function as antimetabolites due to targeting nucleobases and nucleosides, which serve as precursors in the cellular metabolism of nucleic acids. Multiple nucleoside analogues have been employed in the area of cancer therapeutics. These derivatives hinder the activity of ribonucleotide reductase, impeding cellular synthesis of DNA and apoptosis or senescence induction in cancer cells [3–5]. For instance, Pentostatin, Cladribine, and Fludarabine were approved for clinical use in hematological malignancies (Figure 1) [6–8]. Fludarabine, a purine derivative employed in chronic lymphocytic leukaemia (CLL) treatment, exhibits favorable effects on melanoma, breast, and colon carcinoma [9–11]. Investigating different fludarabine complexes, platinum variants displayed enhanced cytotoxicity, particularly against cisplatin-resistant cells, B-cell lymphoma, and CLL, attributed to their ten-fold higher cellular uptake. Notably, platinum complexes exhibited greater selectivity for cancer cells over nonmalignant ones, contrasting with the interaction pattern of palladium complexes with isolated Calf thymus DNA [12]. Moreover, In preclinical studies, cladribine, a purine analogue, has demonstrated the ability to enhance the intracellular uptake of cytarabine in leukemic blasts, suggesting a potential synergistic effect between cladribine and cytarabine [13]. The utilization of cladribine as adjuvant therapy has been a focus of extensive research by the Polish Adult Leukemia Group, conducting several of the most significant studies on the subject [14,15].

Another notable example is Gemcitabine, which has demonstrated success in lung cancer treatment (Figure 1). This drug enters the cell through nucleoside transporters, gets activated by deoxycytidine kinase, and subsequently incorporates itself into cellular genetic material. Gemcitabine exhibits efficacy against various other cancer types, including ovarian cancer (when used alongside triapine or hydroxyurea) [16]. Furthermore, the use of gemcitabine in conjunction with lobaplatin interventional therapy was shown to have the potential to enhance the cure rate of locally advanced cervical cancer (LACC) by decreasing the levels of VEGF and MMP-9 in patients’ serum [17]. Additionally, 5-Fluorouracil (5-FU) serves as another widely employed therapeutic agent for cancer. Although significant advancements have been made in enhancing its anticancer activity over the years, drug resistance remains a significant impediment associated with this molecule (Figure 1) [18].

In this study, we aimed the design of newly synthesized compounds containing a phenyl at the C-8 of the purine structure. These novel derivatives were subsequently generated to produce a fresh series of 6-(substituted phenyl piperazine)-8-(4-phenoxyphenyl)-9-cyclopentyl purine derivatives (5–11), and their biological activities were evaluated against different human liver, colon, and breast cancer cells.

## 2. Results and discussion

### 2.1. Chemistry

4,6-Dichloro-5-nitropyrimidine (1) was used as the starting compound for the synthesis of 6-(substituted phenyl piperazine)-8-(4-substituted phenyl)-9-cyclopentylpurine derivatives (5–11) (Scheme). 4,6-dichloropyrimidine-5-amine (2) was obtained by reduction of 4,6-dichloro-5-nitropyrimidine (1) in the presence of tin (II) chloride (SnCl_2_) in anhydrous medium (in step “a”).

Compound 3 is intended to be obtained by the reaction of 2 and cyclopentylamine in the presence of NEt_3_ via nucleophilic aromatic substitution reaction (in step “b”). By heating pyrimidine derivatives and cyclopentyl amine and 2 in ethanol at 125 °C in a sealed tube, the targeted compounds were reached as a result of nucleophilic aromatic substitution.

In the next step “c”, thanks to the free amine group in the structure of compound (3), when treated with 4-phenoxybenzaldehyde under p-TSA catalysis, a cyclization was obtained as a result of a second addition to the corresponding purine ring (4) on the corresponding imine structures. As a result of the reaction of 3 with 4-phenoxybenzaldehyde in the presence of p-TSA in DMF at 80 °C, compound 4 was obtained.

In the last step “d”, 6,8,9-trisubstituted purine analogs (5–11) were easily prepared in high yield (91%–95%). The target compounds (5–11) were obtained by nucleophilic aromatic substitution with substituted piperazines (Scheme).

As a result, seven new purine derivatives were synthesized, and their purity checks were controlled by TLC and melting point determination. Their chemical structures were characterized by using spectroscopic techniques such as ^1^H, ^13^C NMR, mass, and elemental analyses.

### 2.2. Biological evaluation

**Figure 2:**
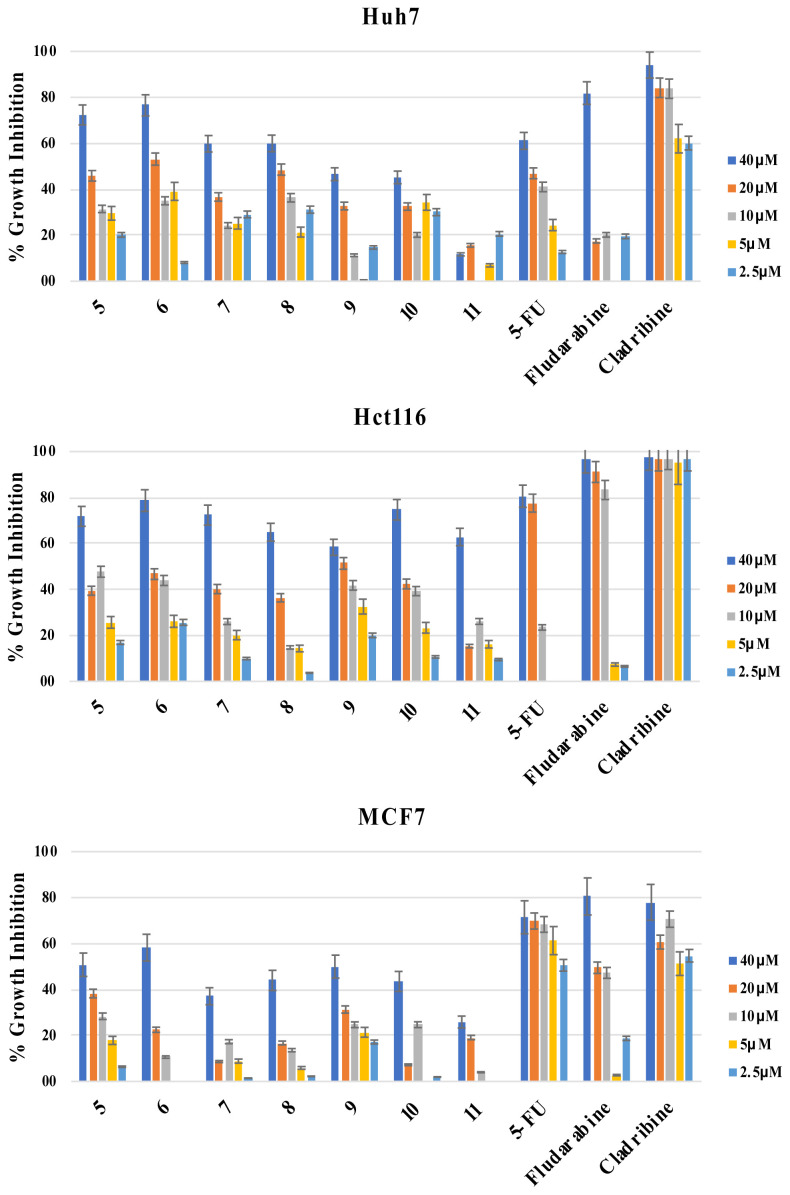
% Growth Inhibition of compounds 5–11 on cancer cells. Cell lines were treated with the compounds for 72 h in triplicate with increasing concentrations (40μM–2.5μM). NCI-SRB analysis was executed to explore the effect of the compounds on cellular growth.

The newly generated purine analogs (5–11) were subjected to in vitro cytotoxicity analysis using the SRB assay, performed in triplicate, against liver, breast, and colon (Huh7, MCF7, and HCT116 respectively) cancer cell lines. The purine compounds were tested at five different concentrations, ranging from 40μM to 2.5μM, over a 72-h period. Positive controls such as Fludarabine, Cladribine, and 5-Fluorouracil (5-FU) were included (Table), (Figure 2).

Among the analogs that contain substituted piperazine group at the C-6 position (5–11), specifically, the 4-methylphenyl substituted piperazine analog (6) displayed lower IC_50_ values compared to clinical positive controls 5-FU and Fludarabine, both in the micromolar concentration range. Compound 6 demonstrated superior cytotoxic activity against Huh7 cells (14.2 μM) compared to 5-FU (30.6 μM) and Fludarabine (28.4 μM). Additionally, the nonsubstituted and methoxyphenyl analogs, 5 (17.9 μM) and 8 (23.6 μM) exhibited higher cytotoxic activities than 5-FU (30.6 μM) and the clinically used nucleoside drug Fludarabine (28.4 μM) on Huh7 cells.

## 3. Conclusion

This research involved creating new purine compounds that had a phenyl group attached to the C-8 position of the purine ring. These compounds were synthesized as a novel set of derivatives called 6-(substituted phenyl piperazine)-8-(4-phenoxyphenyl)-9-cyclopentyl purine derivatives (5–11). The researchers then tested the biotoxic effects of these compounds against various types of cancer cells (including liver, colon, and breast cancer) and the obtained cytotoxicity values were compared to the activity of 5-Fluorouracil (5-FU), Fludarabine, and Cladribine, three clinically used anticancer agents that are frequently used as positive controls in cytotoxicity assays. The results revealed that 5 and 6 exhibited auspicious cytotoxic activity on liver cancer cells with lower IC_50_ values than clinical reference drugs 5-FU and Fludarabine. These results indicated that by using these molecules as scaffolds, new compounds can be synthesized with improved performance for future perspectives.

## 4. Experimental

### 4.1. General

To determine the melting points, an Electrothermal 9100 capillary melting point apparatus was employed. NMR spectra were recorded using a VARIAN Mercury 400 FT-NMR spectrometer operating at frequencies of 400 MHz for ^1^H and 100.6 MHz for ^13^C. TMS served as the internal standard for both ^1^H NMR and ^13^C NMR spectra, with chemical shifts given in ppm and coupling constants in Hz. Mass spectra were obtained using the ESI+ method on a Waters Micro-mass ZQ instrument. Elemental analyses (C, H, N) were conducted on a Leco CHNS 932 instrument, and the determined values were within ±0.4% of the theoretical values. Silica gel 60 (particle size: 40–63mm) was employed for column chromatography. Chemical reagents obtained from reputable suppliers such as Merck, Fluka, Sigma, and Aldrich were utilized for the synthesis process.

### 4.2. Chemistry experimental procedures

5-Amino-4,6-dichloropyrimidine (2) was synthesized according to the reduction methodology reported in the literature [19].

To a solution of 5-Amino-4,6-dichloropyrimidine (2) (6.00 g, 193.98 mmol) in 100 mL ethanol, SnCl_2_.2H_2_O (27.90 g, 123.70 mmol) was sequentially added and refluxed for 2 h. Upon completion of the reaction, as detected by TLC, the reaction mixture was concentrated under vacuum. The reaction mixture was quenched using an aqueous saturated solution of NaHCO_3_ until pH = 8. The water phase was extracted with EtOAc. The organic layers were combined and dried over Na_2_SO_4_, followed by concentration under vacuum. As a result, compound 2 was found to be (4.97 g, 98% yield) as a yellow solid. mp = 149–151 °C. ^1^H NMR (400 MHz, CDCl_3_) δ 8.19 (s, 1H), 4,52 (bs, 2H). ^13^C NMR (100 MHz, CDCl_3_) δ 135.90, 144.19, 145.96.

#### 4.2.1. 6-Chloro-N^4^-cyclopentylpyrimidine-4,5-diamine (3)

To a solution of 2 (1.0 g, 6.09 mmol) in MeOH, cyclopentylamine was added (2.38 g, 28.08 mmol). Then the mixture was heated in a sealed tube at 125 °C for 6 h. Afterward, the mixture was concentrated under vacuum, and the crude product was subjected to purification using silica gel column chromatography (10:1 CH_2_Cl_2_: MeOH). As a result, compound 3 was found to be (1.15 g, 89% yield) as a light-yellow solid. mp = 140–142 °C. ^1^H NMR (400 MHz, CDCl_3_) δ 1.40–1.48 (m, 2H), 1,61–1,73 (m, 4H), 2.06–2.13 (m, 2H), 3.43 (bs, 2H) 4.34–4.39 (m, 1H), 4.93 (bs, 1H), 8.06 (s, 1H). ^13^C NMR (100 MHz, CDCl_3_) δ 23.71, 33.30, 53.15, 121.56, 142.88, 149.81, 155.04. MS (ESI+) m/e: 213.1 (100%) [M + H]^+^.

#### 4.2.2. 6-Chloro-9-cyclopentyl-8-(4-phenoxyphenyl)-9H-purine (4)

To a solution of compound 3 (4.70 mmol) in 10 mL DMF, benzaldehyde derivatives (9,40 mmol) and p-TsOH (0.17 g, 0.94 mmol) were sequentially added and stirred overnight at 80 °C. Upon completion of the reaction, as detected by TLC, the reaction mixture was concentrated under vacuum. The reaction mixture was quenched using an aqueous saturated solution of NH_4_Cl, and the water phase was extracted with DCM. The organic layers were combined and dried over Na_2_SO_4_, followed by concentration under vacuum. The crude products were subjected to purification using silica gel column chromatography (1:5 EtOAc:Hexane) to afford 4 (0.97 g, 53% yield). mp = 146–148 °C. ^1^H NMR (400 MHz, CDCl_3_) δ 1.62–1.75 (m, 2H), 2.0–2.20 (m, 4H), 2.50–2.62 (m, 2H), 4.80–4.90 (m, 1H), 7.10 (d, 2H), 7.14 (d, 2H), 7.20 (t, 1H), 7.41 (t, 2H), 7.67 (d, 2H), 8.70 (s, 1H). ^13^C NMR (100 MHz, CDCl_3_) δ 24.95, 30.98, 58.51 (C-cyclopentyl), 118.29, 119.91, 123.48, 124.44, 130.05, 131.31, 131.97, 150.02 (C-phenyl), 150.63, 152.90, 155.81, 156.34, 160.04 (C-purine). MS (ESI+) *m/e*: 391.3 (100%) [M + H]^+^, 393.0 35(%) [M + 2]^+^. Anal. calcd for C_22_H_19_ClN_4_O.0.2H_2_O; C, 66.98; H, 4.96; N, 14.20. Found: C, 66.83; H, 4.80; N, 14.29.

#### 4.2.3. General procedure for preparation of compounds 5-11

To the solution of compound 4 (1 equiv.) in absolute EtOH (10 mL) 4-substituted piperazine derivatives were added (1 equiv.) and Et_3_N (3 equiv.). The resulting solution was then refluxed at 80–90 °C. After 6 h reflux period, the reaction mixture was concentrated. The crude product was subjected to purification using silica gel column chromatography (1:10/1:5 EtOAc:Hexane) to afford 5–11.

##### 4.2.3.1. 9-Cyclopentyl-8-(4-phenoxyphenyl)-6-(4-phenylpiperazin-1-yl)-9H-purine (5)

The above procedure was followed with 1-phenylpiperazine to yield 5 (0.113 g, 95% yield). mp = 173–175 °C. ^1^H NMR (400 MHz, CDCl_3_) δ 1.59–1.68 (m, 2H), 1.93–2.16 (m, 4H), 2.55–2.67 (m, 2H), 3.32 (t, 4H), 4.49 (br s, 4H), 4.70–4.78 (m, 1H), 6.88 (t, 1H), 6.97 (d, 2H), 7.14 (t, 4H), 7.19 (t, 1H), 7.28 (t, 2H), 7.40 (t, 2H), 7.61 (d, 2H), 8.36 (s, 1H). ^13^C NMR (100 MHz, CDCl_3_) δ 24.73, 30.64 (C-cyclopentyl), 45.09, 49.64 (CH_2_-piperazine), 57.89 (C-cyclopentyl), 116.44, 118.27, 119.79, 120.12, 120.71, 124.20, 125.13, 129.18, 129.98, 131.10, 149.48, 151.21 (C-phenyl), 151.34, 152.20, 153.68, 156.11, 159.13 (C-purine). MS (ESI+) m/e: 517.2 (100%) [M + H]^+^. Anal. calcd for C_32_H_32_N_6_O.0.35H_2_O; C, 73.49; H, 6.30; N, 16.07. Found: C, 73.76; H, 6.50; N, 15.91.

##### 4.2.3.2. 9-Cyclopentyl-8-(4-phenoxyphenyl)-6-(4-(p-tolyl)piperazin-1-yl)-9H-purine (6)

The above procedure was followed with 1-(p-tolyl)piperazine to yield 6 (0.115 g, 94% yield). mp = 170–173 °C. ^1^H NMR (400 MHz, CDCl_3_) δ 1.58–1.70 (m, 2H), 1.92–2.15 (m, 4H), 2.27 (s, 3H), 2.55–2.67 (m, 2H), 3.25 (t, 4H), 4.48 (br s, 4H), 4.69–4.78 (m, 1H), 6.89 (d, 2H), 7.08–7.14 (m, 6H), 7.18 (t, 1H), 7.40 (t, 2H), 7.60 (d, 2H), 8.36 (s, 1H). ^13^C NMR (100 MHz, CDCl_3_) δ 20.43 (–CH_3_), 24.73, 30.63 (C-cyclopentyl), 45.11, 50.23 (CH_2_-piperazin), 57.88 (C-cyclopentyl), 116.82, 118.26, 119.78, 120.69, 124.18, 125.14, 129.70, 129.98, 131.10, 149.25, 149.43 (C-phenyl), 151.21, 152.19, 153.68, 156.11, 159.11 (C-purine). MS (ESI+) m/e: 531.2 (100%) [M + H]^+^. Anal. calcd for C_33_H_34_N_6_O.0.3H_2_O; C, 73.93; H, 6.50; N, 15.67. Found: C, 74.22; H, 6.56; N, 15.62.

##### 4.2.3.3. 9-Cyclopentyl-8-(4-phenoxyphenyl)-6-(4-(4-(trifluoromethyl)phenyl) piperazin-1-yl)-9H-purine (7)

The above procedure was followed with 1-(4-(trifluoromethyl)phenyl)piperazine to yield **7** (0.126 g, 94% yield). mp = 172–174 °C. ^1^H NMR (400 MHz, CDCl_3_) δ 1.59–1.70 (m, 2H), 1.92–2.16 (m, 4H), 2.54–2.67 (m, 2H), 3.41 (t, 4H), 4.49 (br s, 4H), 4.70–4.78 (m, 1H), 6.96 (d, 2H), 7.12 (t, 4H), 7.19 (t, 1H), 7.40 (t, 2H), 7.50 (d, 2H), 7.60 (d, 2H), 8.37 (s, 1H). ^13^C NMR (100 MHz, CDCl_3_) δ 24.74, 30.65 (C-cyclopentyl), 44.77, 48.29 (CH_2_-piperazin), 57.91 (C-cyclopentyl), 114.77, 118.26, 119.81, 120.69, 123.32, 124.24, 125.03, 126.01, 126.45 (q) (–CF_3_), 129.99, 131.07, 149.65, 151.19 (C- phenyl), 152.23, 153.28, 153.59, 156.07, 159.19 (C-purine). MS (ESI+) m/e: 585.2 (100%) [M + H]^+^. Anal. calcd for C_33_H_31_F_3_N_6_O.0.5H_2_O; C, 66.76; H, 5.43; N, 14.16. Found: C, 66.52; H, 5.20; N, 13.95.

##### 4.2.3.4. 9-Cyclopentyl-6-(4-(4-methoxyphenyl)piperazin-1-yl)-8-(4-phenoxy phenyl)-9H-purine (8)

The above procedure was followed with 1-(4-methoxyphenyl)piperazine to yield 8 (0.115 g, 91% yield). mp = 160–162 °C. ^1^H NMR (400 MHz, CDCl_3_) δ 1.58–1.70 (m, 2H), 1.92–2.15 (m, 4H), 2.55–2.66 (m, 2H), 3.19 (t, 4H), 3.77 (s, 3H), 4.48 (br s, 4H), 4.69–4.77 (m, 1H), 6.85 (d, 2H), 6.95 (d, 2H), 7.11 (t, 4H), 7.18 (t, 1H), 7.40 (t, 2H), 7.60 (d, 2H), 8.36 (s, 1H). ^13^C NMR (100 MHz, CDCl_3_) δ 24.72, 30.62 (C-cyclopentyl), 45.19, 51.16 (CH_2_-piperazin), 55.54 (–OCH_3_), 57.88 (C-cyclopentyl), 114.48, 118.26, 118.65, 119.78, 120.69, 124.19, 125.14, 129.98, 131.10, 145.71, 149.42, 151.21 (C-phenyl), 152.19, 153.69, 154.12, 156.11, 159.11 (C-purine). MS (ESI+) m/e: 547.3 (100%) [M + H]^+^. Anal. calcd for C_33_H_34_N_6_O_2_.0.1H_2_O; C, 72.26; H, 6.29; N, 15.32. Found: C, 71.96; H, 6.39; N, 15.15.

##### 4.2.3.5. 9-Cyclopentyl-6-(4-(4-fluorophenyl)piperazin-1-yl)-8-(4-phenoxy phenyl)-9H-purine (9)

The above procedure was followed with 1-(4-fluorophenyl)piperazine to yield 9 (0.113 g, 92% yield). mp = 145–147 °C. ^1^H NMR (400 MHz, CDCl_3_) δ 1.60–1.70 (m, 2H), 1.92–2.16 (m, 4H), 2.54–2.66 (m, 2H), 3.22 (t, 4H), 4.48 (br s, 4H), 4.69–4.78 (m, 1H), 6.90–7.0 (m, 4H), 7.11 (t, 4H), 7.18 (t, 1H), 7.40 (t, 2H), 7.60 (d, 2H), 8.36 (s, 1H). ^13^C NMR (100 MHz, CDCl_3_) δ 24.73, 30.63 (C-cyclopentyl l), 45.09, 50.67 (CH_2_-piperazin), 57.89 (C-cyclopentyl), 115.61 (d), 118.26, 119.80, 120.70, 124.21, 125.09, 129.98, 131.08, 148.02 (d), 149.51, 151.19, 152.21 (C-phenyl), 153.66, 156.08, 156.22, 158.60, 159.15 (C-purine). MS (ESI+) m/e: 535.2 (100%) [M + H]^+^. Anal. calcd for C_32_H_31_FN_6_O; C, 71.89; H, 5.84; N, 15.72. Found: C, 71.70 H, 6.00; N, 15.35.

##### 4.2.3.6. 6-(4-(4-Chlorophenyl)piperazin-1-yl)-9-cyclopentyl-8-(4-phenoxy phenyl)-9H-purine (10)

The above procedure was followed with 1-(4-chlorophenyl)piperazine to yield 10 (0.117 g, 92% yield). mp = 168–169 °C. ^1^H NMR (400 MHz, CDCl_3_) δ 1.57–1.70 (m, 2H), 1.92–2.15 (m, 4H), 2.54–2.67 (m, 2H), 3.27 (t, 4H), 4.48 (br s, 4H), 4.69–4.78 (m, 1H), 6.88 (d, 2H), 7.09–7.23 (m, 7H), 7.40 (t, 2H), 7.60 (d, 2H), 8.36 (s, 1H). ^13^C NMR (100 MHz, CDCl_3_) δ 24.73, 30.64 (C-cyclopentyl), 44.93, 49.61 (CH_2_-piperazin), 57.90 (C-cyclopentyl), 117.60, 118.26, 119.80, 124.21, 124.97, 125.07, 129.03, 129.98, 131.08, 149.56, 149.95 (C-phenyl), 151.19, 152.21, 153.62, 156.08, 159.15 (C-purine). MS (ESI+) m/e: 551.2 (100%) [M]^+^, 553.3 (36%) [M + 2]^+^. Anal. calcd for C_32_H_31_ClN_6_O.0.05H_2_O; C, 69.63; H, 5.68; N, 15.22. Found: C, 69.91; H, 5.88; N, 14.89.

##### 4.2.3.7. 9-Cyclopentyl-6-(4-(3,4-dichlorophenyl)piperazin-1-yl)-8-(4-phenoxy phenyl)-9H-purine (11)

The above procedure was followed with 1-(3,4-dichlorophenyl)piperazine to yield 11 (0.124 g, 92% yield). mp = 178–179 °C. ^1^H NMR (400 MHz, CDCl_3_) δ 1.58–1.70 (m, 2H), 1.92–2.16 (m, 4H), 2.54–2.65 (m, 2H), 3.28 (t, 4H), 4.47 (br s, 4H), 4.70–4.78 (m, 1H), 6.78 (dd, 1H), 6.99 (d, 1H), 7.09–7.30 (m, 6H), 7.40 (t, 2H), 7.60 (d, 2H), 8.36 (s, 1H). ^13^C NMR (100 MHz, CDCl_3_) δ 24.74, 30.65 (C-cyclopentyl), 44.76, 49.06 (CH_2_-piperazin), 57.91 (C-cyclopentyl), 115.63, 117.56, 118.26, 119.80, 120.72, 122.59, 124.23, 125.01, 129.99, 130.50, 131.07, 132.86, 149.60, 150.64 (C-phenyl), 151.18, 152.0, 153.46, 156.04, 159.18 (C-purine).MS (ESI+) *m/e*: 585.3 (100%) [M]^+^, 587.2 (65%) [M + 2]^+^, 589.3 (12%) [M + 4]^+^. Anal. calcd for C_32_H_30_Cl_2_N_6_O; C, 65.64; H, 5.16; N, 14.35. Found: C, 65.60; H, 4.96; N, 14.14.

All NMR and Mass spectra are provided in the Supplementary Information.

### 4.3. Cytotoxicity

#### 4.3.1. Cell culture

The human cancer cell lines were cultured in standard Dulbecco’s Modified Eagle Medium (DMEM) supplemented with 10% fetal bovine serum (FBS) and 1% penicillin.

#### 4.3.2. NCI-60 Sulphorhodamine B (SRB) assay [20]

Cancer cells, (2000–5000 cells per well) were seeded into 96-well plates. After 24 h of incubation, the cells were exposed to escalating concentrations of the compounds (2.5μM to 40μM) for a duration of 72 h. Then the cells were fixed using 10% ice-cold trichloroacetic acid (TCA) in the dark at + 4 °C for one h. The plates were stained with a solution of 0.4% sulphorhodamine B (SRB) in a 1% acetic acid solution. The bound SRB stain was dissolved to measure the absorbance using a 10 mM Tris-Base solution, and the optical density (OD) values were recorded at a wavelength of 515 nm.

## Supplementary information: Spectral data

Figure S1 ^1^H NMR spectrum of Compound 4.

Figure S2 ^13^C NMR spectrum of Compound 4.

Figure S3 Mass spectrum of Compound 4.

Figure S4 ^1^H NMR spectrum of Compound 5.

Figure S5 ^13^C NMR spectrum of Compound 5.

Figure S6 Mass spectrum of Compound 5.

Figure S7 ^1^H NMR spectrum of Compound 6.

Figure S8 ^13^C NMR spectrum of Compound 6.

Figure S9 Mass spectrum of Compound 6.

Figure S10 ^1^H NMR spectrum of Compound 7.

Figure S11 ^13^C NMR spectrum of Compound 7.

Figure S12 Mass spectrum of Compound 7.

Figure S13 ^1^H NMR spectrum of Compound 8.

Figure S14 ^13^C NMR spectrum of Compound 8.

Figure S15 Mass spectrum of Compound 8.

Figure S16 ^1^H NMR spectrum of Compound 9.

Figure S17 ^13^C NMR spectrum of Compound 9.

Figure S18 Mass spectrum of Compound 9.

Figure S19 ^1^H NMR spectrum of Compound 10.

Figure S20 ^13^C NMR spectrum of Compound 10.

Figure S21 Mass spectrum of Compound 10.

Figure S22 ^1^H NMR spectrum of Compound 11.

Figure S23 ^13^C NMR spectrum of Compound 11.

Figure S24 Mass spectrum of Compound 11.

## Figures and Tables

**Scheme:**
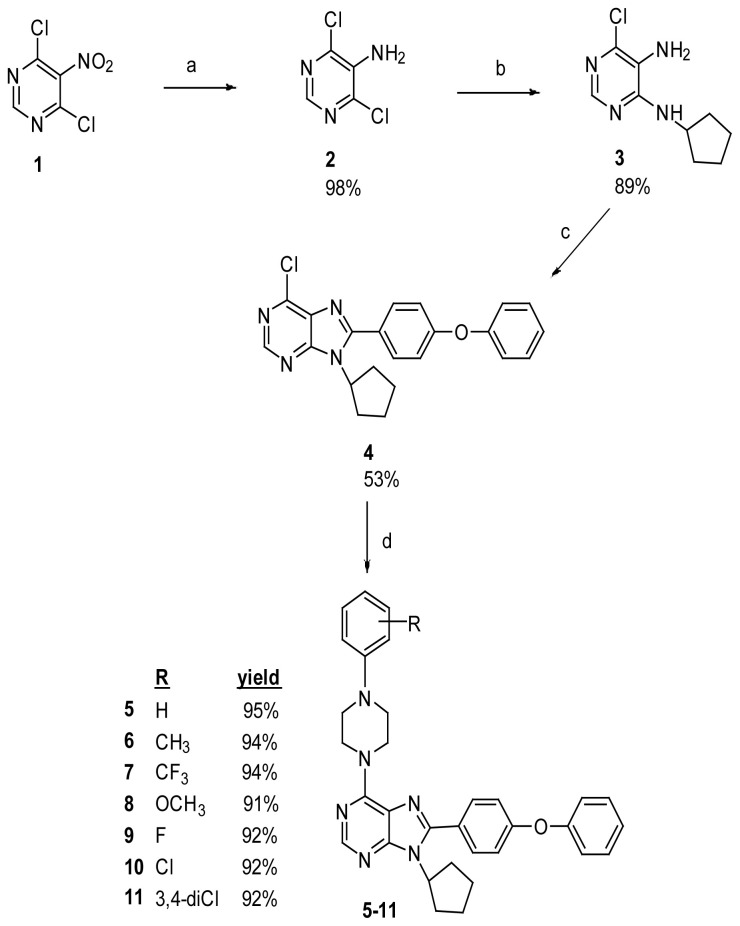
Synthesis of compounds 5–11. Reagents: (a) SnCl_2_.2H_2_O, EtOH, 2; (b) cyclopentyl amine, EtOH, 3; (c) 4-substituted benzaldehydes, p-TsOH, DMF. (d) substituted piperazines, Et_3_N, EtOH.

**Table:** The cytotoxicity of compounds 5–11 was evaluated in vitro.

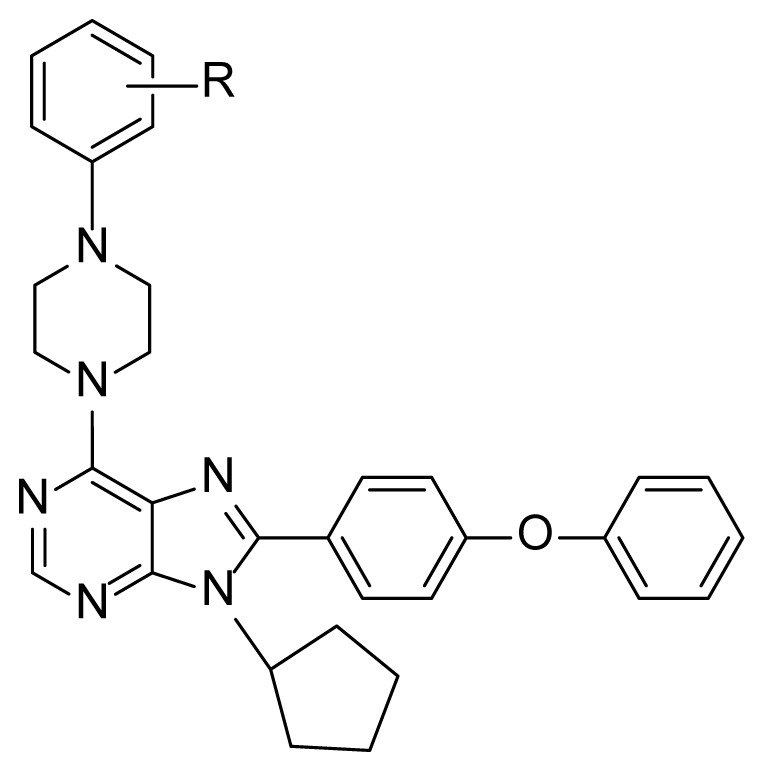				

Compound	R	IC_50_ (μM)		

		HUH7	HCT116	MCF7
**5 (ME69)**	H	17.9 ± 0.9	17.2 ± 1.7	39.6 ± 4.8
**6 (ME70)**	CH_3_	14.2 ± 1.4	13.7 ± 2.7	41.7 ± 3.8
**7 (ME71)**	CF_3_	41.5 ± 8.3	21.8 ± 1.7	NI
**8 (ME72)**	OCH_3_	23.6 ± 7.1	30.4 ± 4	NI
**9 (ME73)**	F	80.1 ± 16.0	19.5 ± 1.0	69.2 ± 11.8
**10 (ME74)**	Cl	NI	17.6 ± 5.3	NI
**11 (ME75)**	3,4-diCl	NI	48.2 ± 9.6	NI
**5-FU**		30.6 ± 1.8	4.1 ± 0.3	3.5 ± 0.7
**Fludarabine**		28.4 ± 19.2	8.0 ± 3.4	15.2 ± 0.1
**Cladribine**		0.9 ± 0.7	<0.1	2.4 ± 2.4

SRB experiment and data were expressed as means of ± SD.

NI: No inhibition

## References

[1] Sung H, Ferlay J, Siegel RL, Laversanne M, Soerjomataram I (2021). Global cancer statistics 2020: Globocan estimates of incidence and mortality worldwide for 36 cancers in 185 countries. CA: A Cancer Journal for Clinicians.

[2] Karran P (2006). Thiopurines, DNA damage, DNA repair and therapy-related cancer. British Medical Bulletin.

[3] Dick JE, Wright JA (1985). On the importance of deoxyribonucleotide pools in the senescence of cultured human diploid fibroblasts. FEBS Letters.

[4] Nakamura J, Kohya N, Kai K, Ohtaka K, Hashiguchi K (2010). Ribonucleotide reductase subunit M1 assessed by quantitative double-fluorescence immunohistochemistry predicts the efficacy of gemcitabine in biliary tract carcinoma. International Journal of Oncology.

[5] Wilson PK, Mulligan SP, Christopherson RI (2004). Metabolic response patterns of nucleotides in B-cell chronic lymphocytic leukaemias to cladribine, fludarabine and deoxycoformycin. Leukemia Research.

[6] Escherich G, Richards S, Stork LC, Vora AJ (2011). Meta-analysis of randomised trials comparing thiopurines in childhood acute lymphoblastic leukaemia. Leukemia.

[7] Parker WB (2009). Enzymology of purine and pyrimidine antimetabolites used in the treatment of cancer. Chemical Reviews.

[8] Robak P, Robak T (2013). Older and new purine nucleoside analogs for patients with acute leukemias. Cancer Treatment Reviews.

[9] Hanauske AR, Von Hoff DD (2021). Clinical development of fludarabine Nucleoside analogs in cancer therapy.

[10] Huang C, Tu Y, Freter CE (2018). Fludarabine-resistance associates with ceramide metabolism and leukemia stem cell development in chronic lymphocytic leukemia. Oncotarget.

[11] Burger JA (2020). Treatment of chronic lymphocytic leukemia. New England Journal of Medicine.

[12] Schleser SW, Krytovych O, Ziegelmeier T, Groß E, Kasparkova J (2023). Palladium and platinum complexes of the antimetabolite fludarabine with vastly enhanced selectivity for tumour over non-malignant cells. Molecules.

[13] Holowiecki J, Grosicki S, Robak T, Kyrcz Krzemien S, Giebel S (2004). Addition of cladribine to daunorubicin and cytarabine increases complete remission rate after a single course of induction treatment in acute myeloid leukemia. Multicenter, phase III study. Leukemia.

[14] Holowiecki J, Grosicki S, Giebel S, Robak T, Kyrcz Krzemien S (2012). Cladribine, but not fludarabine, added to daunorubicin and cytarabine during induction prolongs survival of patients with acute myeloid leukemia: a multicenter, randomized phase III study. Journal of Clinical Oncology.

[15] Michaelis LC (2021). Is there an optimal adjunct therapy to traditional cytotoxic induction?. Best Practice & Research Clinical Haematology.

[16] Muggia F, Diaz I, Peters GJ (2012). Nucleoside and nucleobase analogs in cancer treatment: not only sapacitabine, but also gemcitabine. Expert Opinion on Investigational Drugs.

[17] Yang L, Qiaoli Y (2023). Mechanism of gemcitabine combined with lobaplatin in interventional treatment of locally advanced cervical cancer. Anti-Cancer Drugs.

[18] Kuhn JG (2001). Fluorouracil and the new oral fluorinated pyrimidines. Annals of Pharmacotherapy.

[19] Latli B, Jones PJ, Krishnamurthy D, Senanayake CH (2008). Synthesis of [14C]-,[13C4]-, and [13C4, 15N2]-5-amino-4-iodopyrimidine. Journal of Labelled Compounds and Radiopharmaceuticals.

[20] Shoemaker RH (2006). The NCI60 human tumor cell line anticancer drug screen. Nature Reviews Cancer.

