# Supplementary figures and images for: Exploration of novel 6,8,9-trisubstituted purine analogues: synthesis, in vitro biological evaluation, and their effect on human cancer cells
Source: Turk J Chem. 2023 Dec 4;48(1):108–15. doi: 10.55730/1300-0527.3643 (PMC10965180; doi:10.55730/1300-0527.3643)

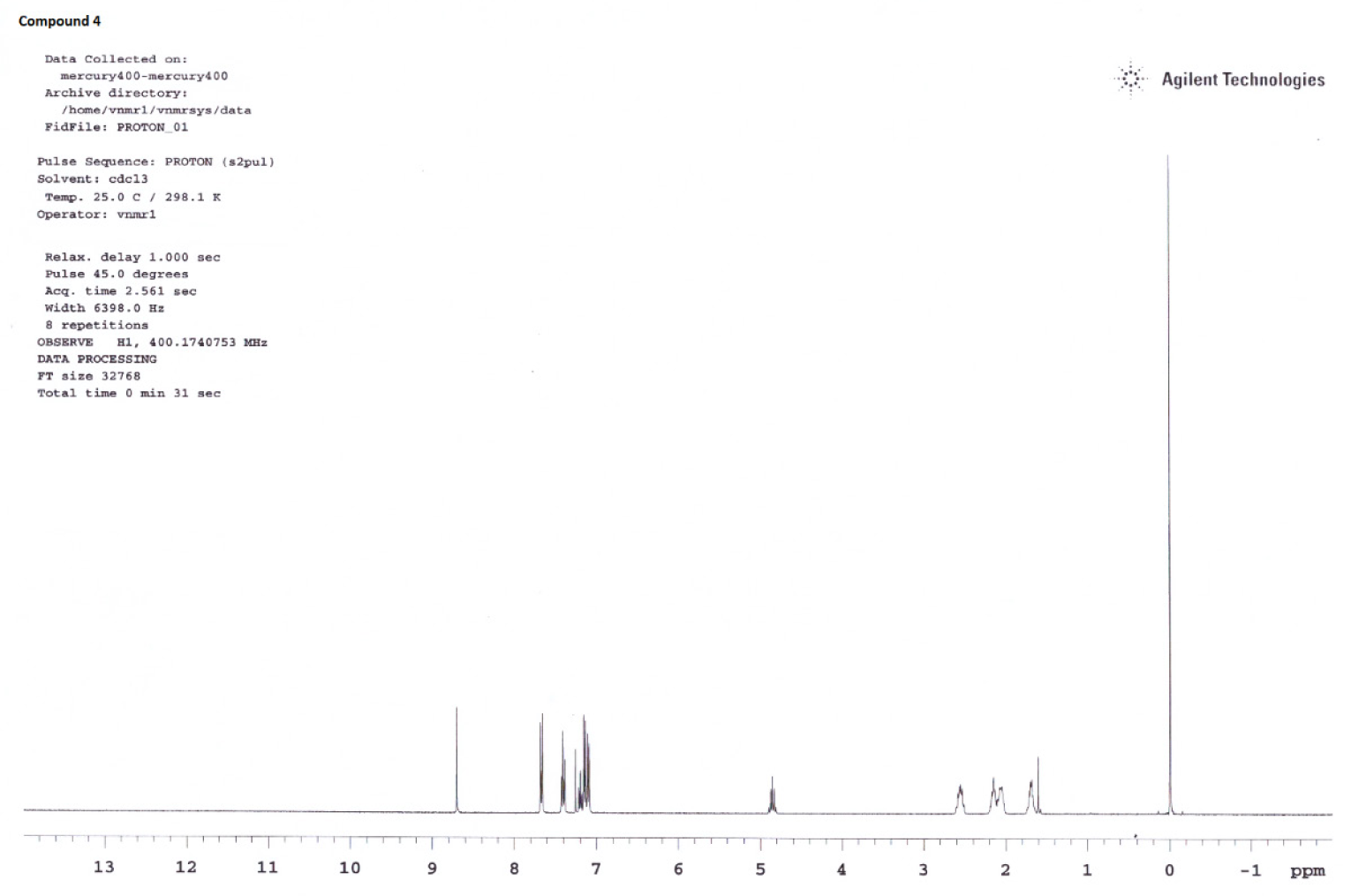

Supplement: Figure S1 — 1H NMR spectrum of Compound 4. [file tjc-48-01-0108s1.tif]

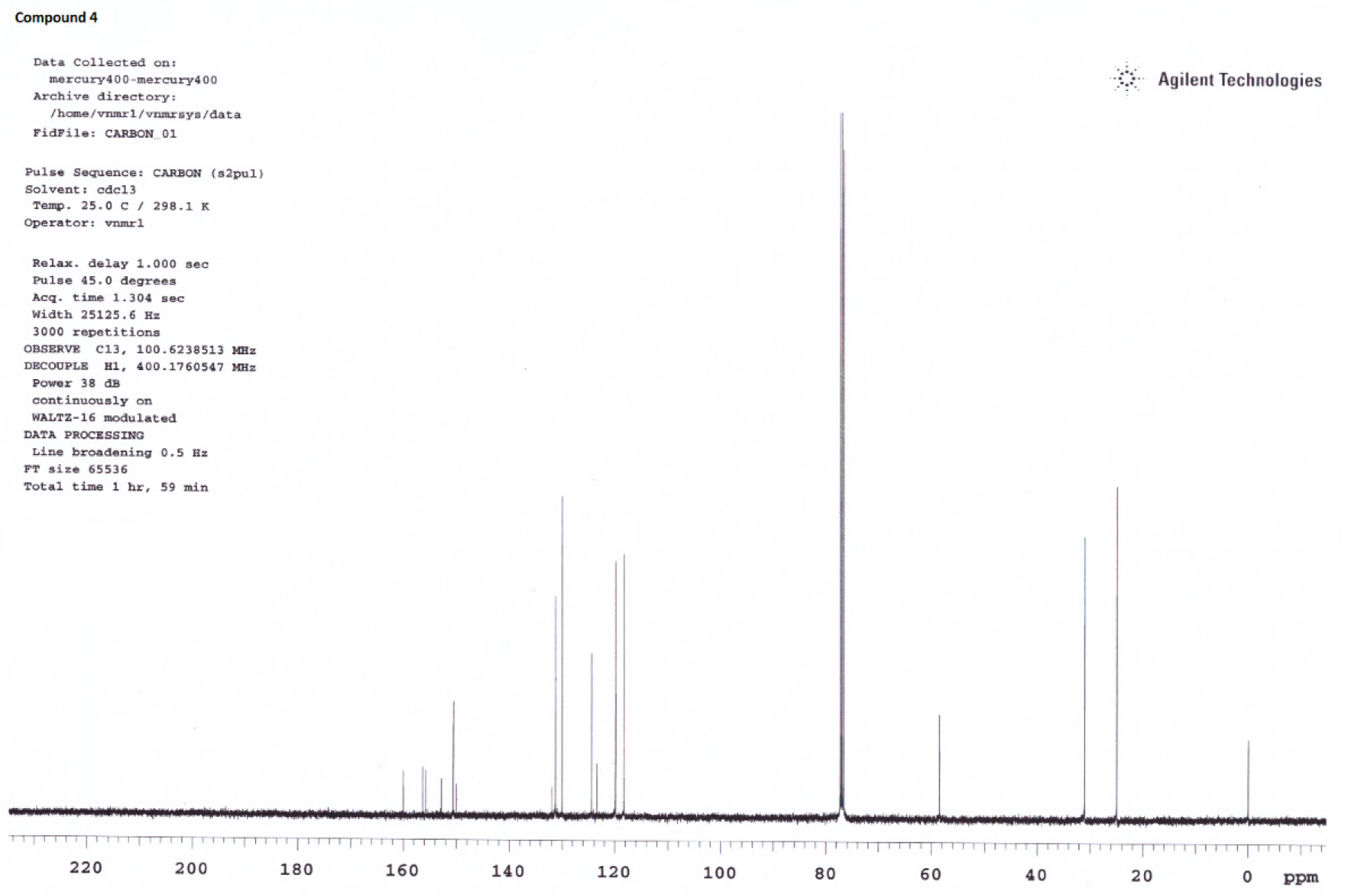

Supplement: Figure S2 — 13C NMR spectrum of Compound 4. [file tjc-48-01-0108s2.tif]

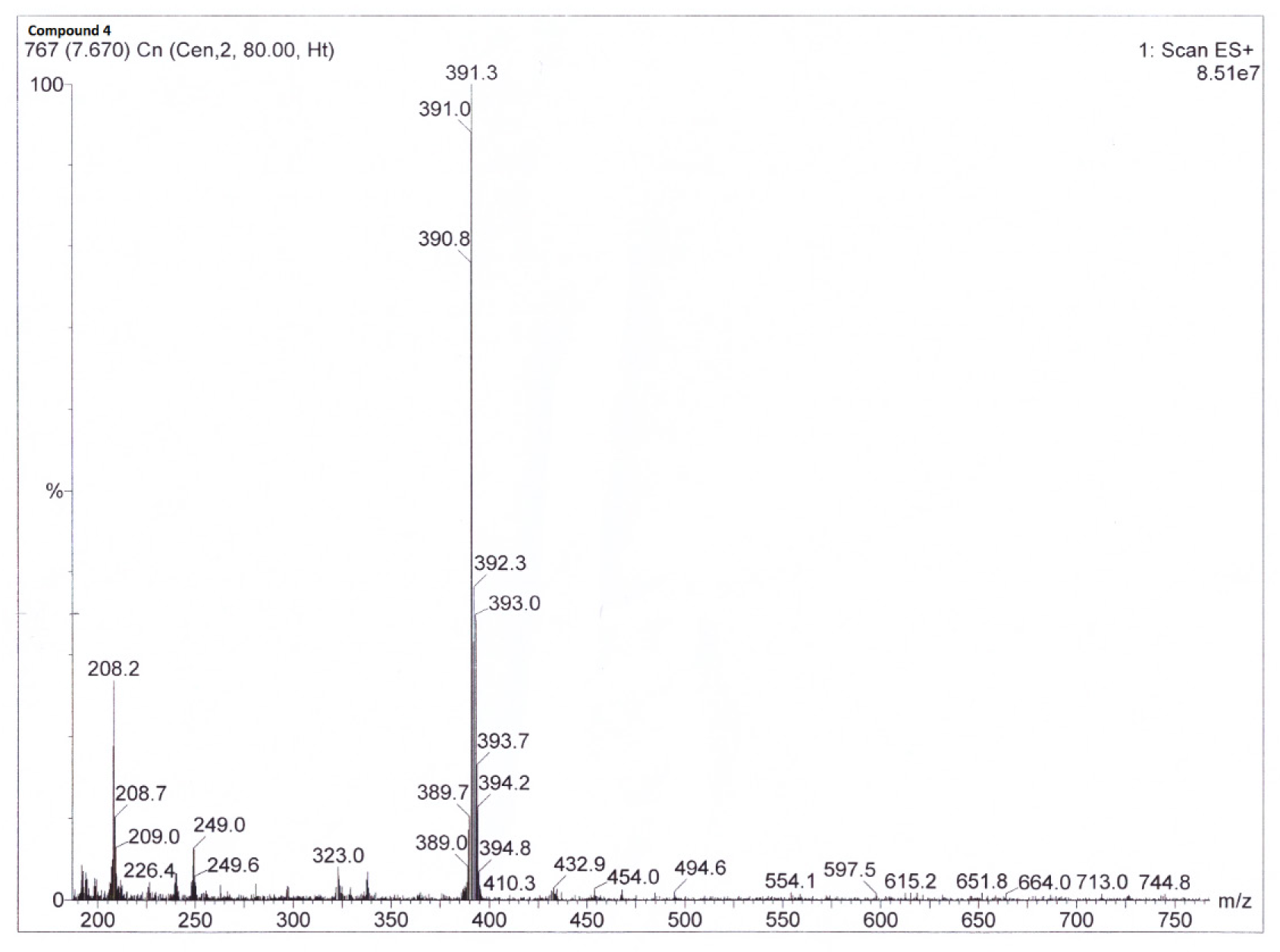

Supplement: Figure S3 — Mass spectrum of Compound 4. [file tjc-48-01-0108s3.tif]

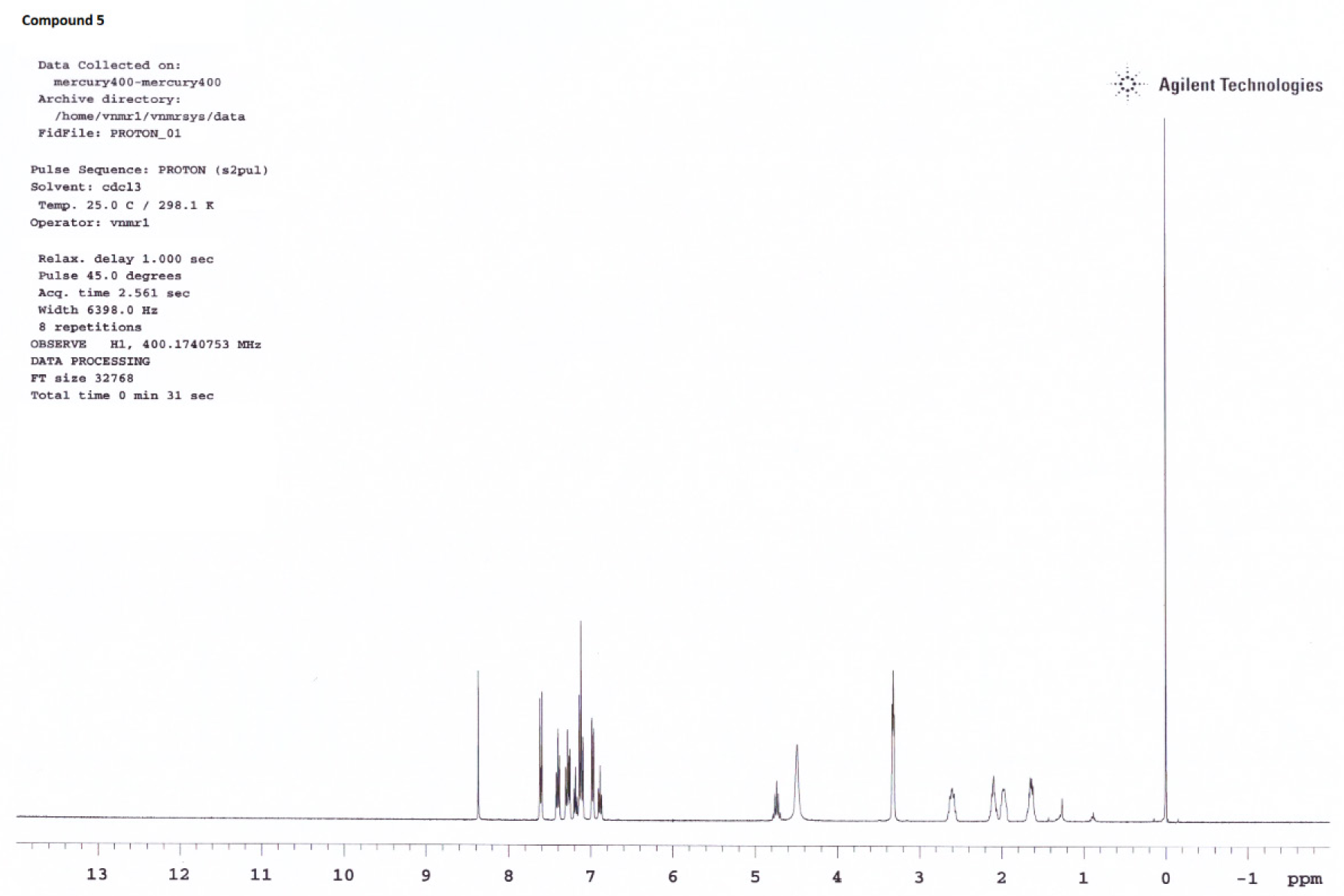

Supplement: Figure S4 — 1H NMR spectrum of Compound 5. [file tjc-48-01-0108s4.tif]

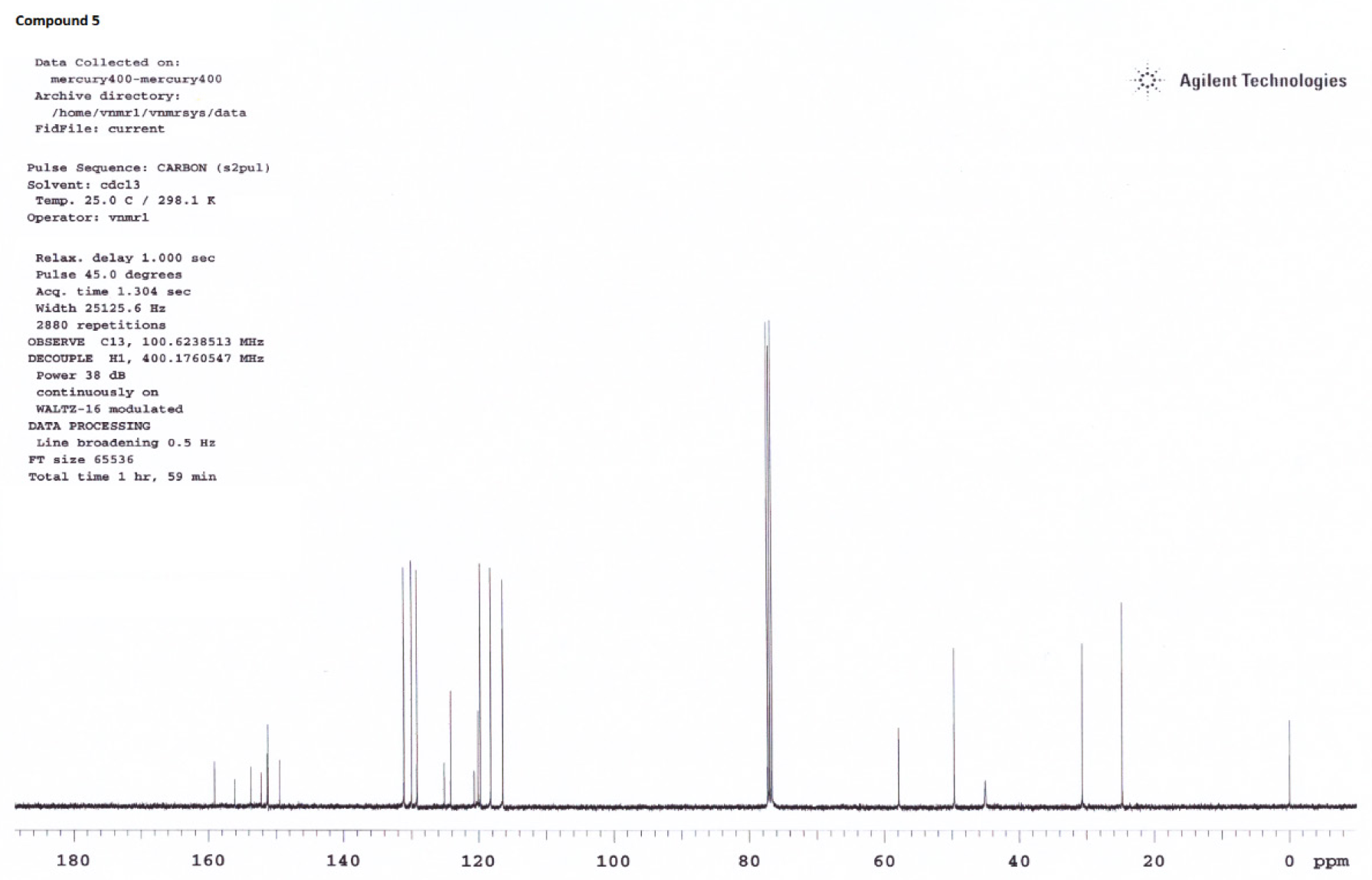

Supplement: Figure S5 — 13C NMR spectrum of Compound 5. [file tjc-48-01-0108s5.tif]

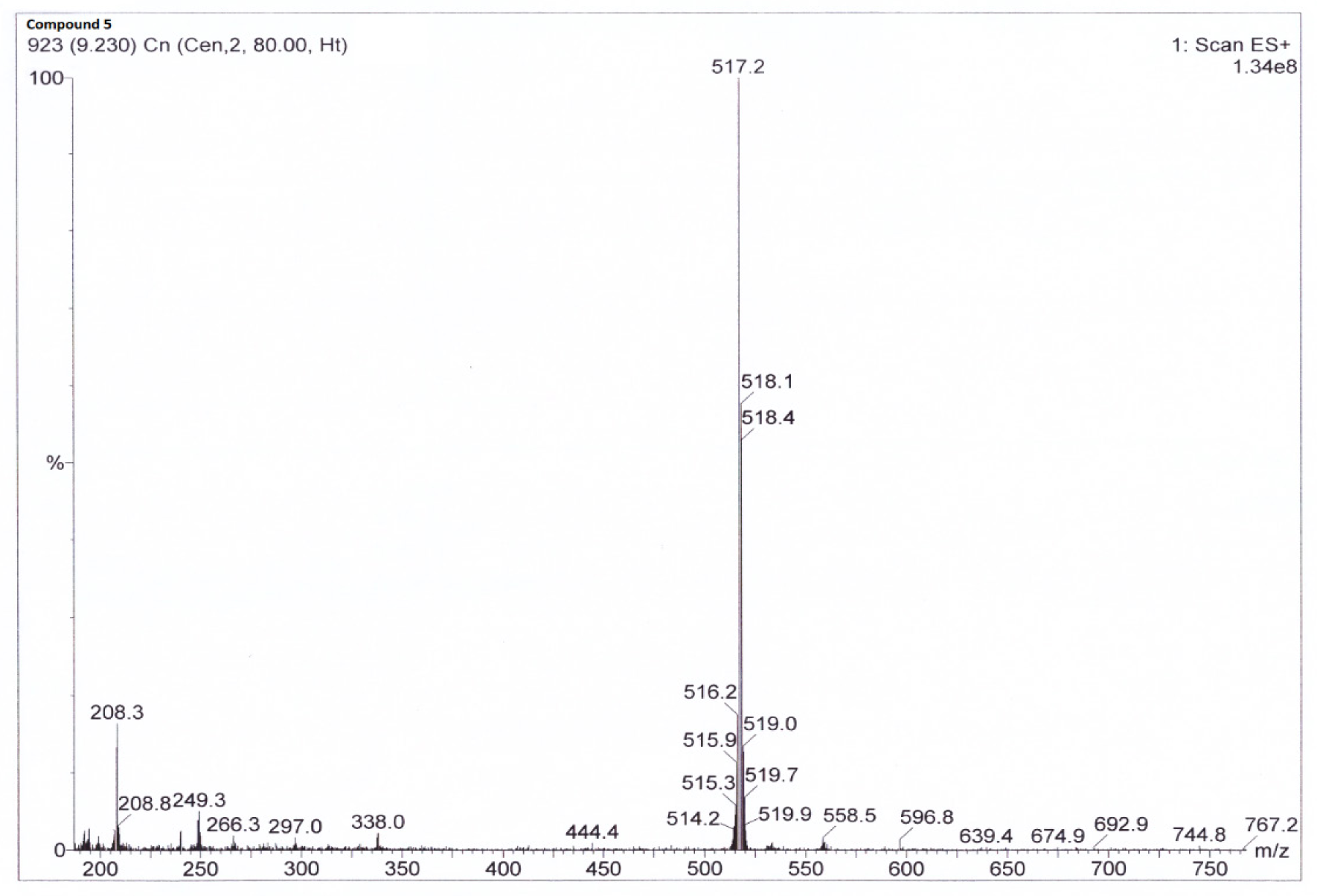

Supplement: Figure S6 — Mass spectrum of Compound 5. [file tjc-48-01-0108s6.tif]

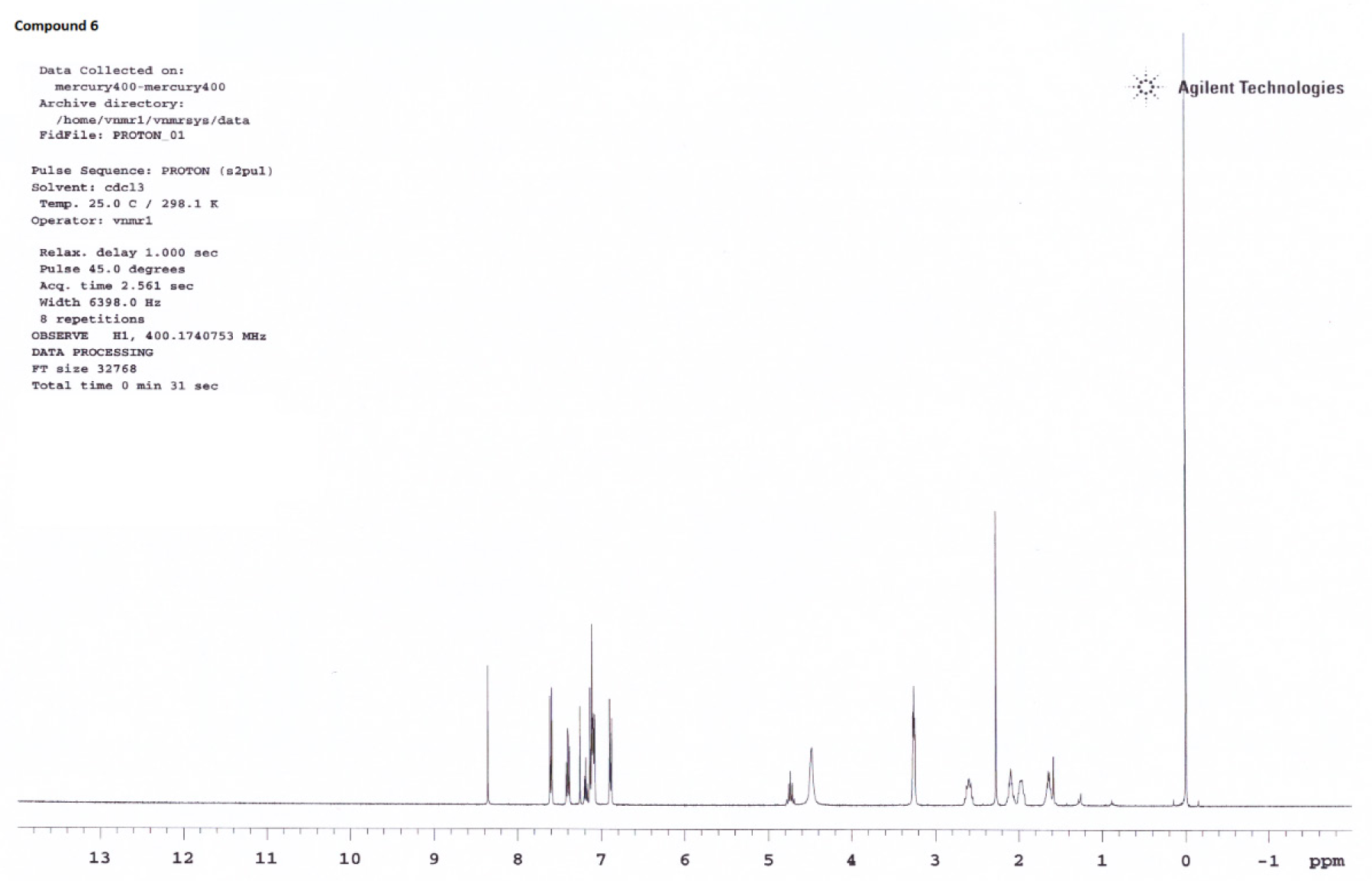

Supplement: Figure S7 — 1H NMR spectrum of Compound 6. [file tjc-48-01-0108s7.tif]

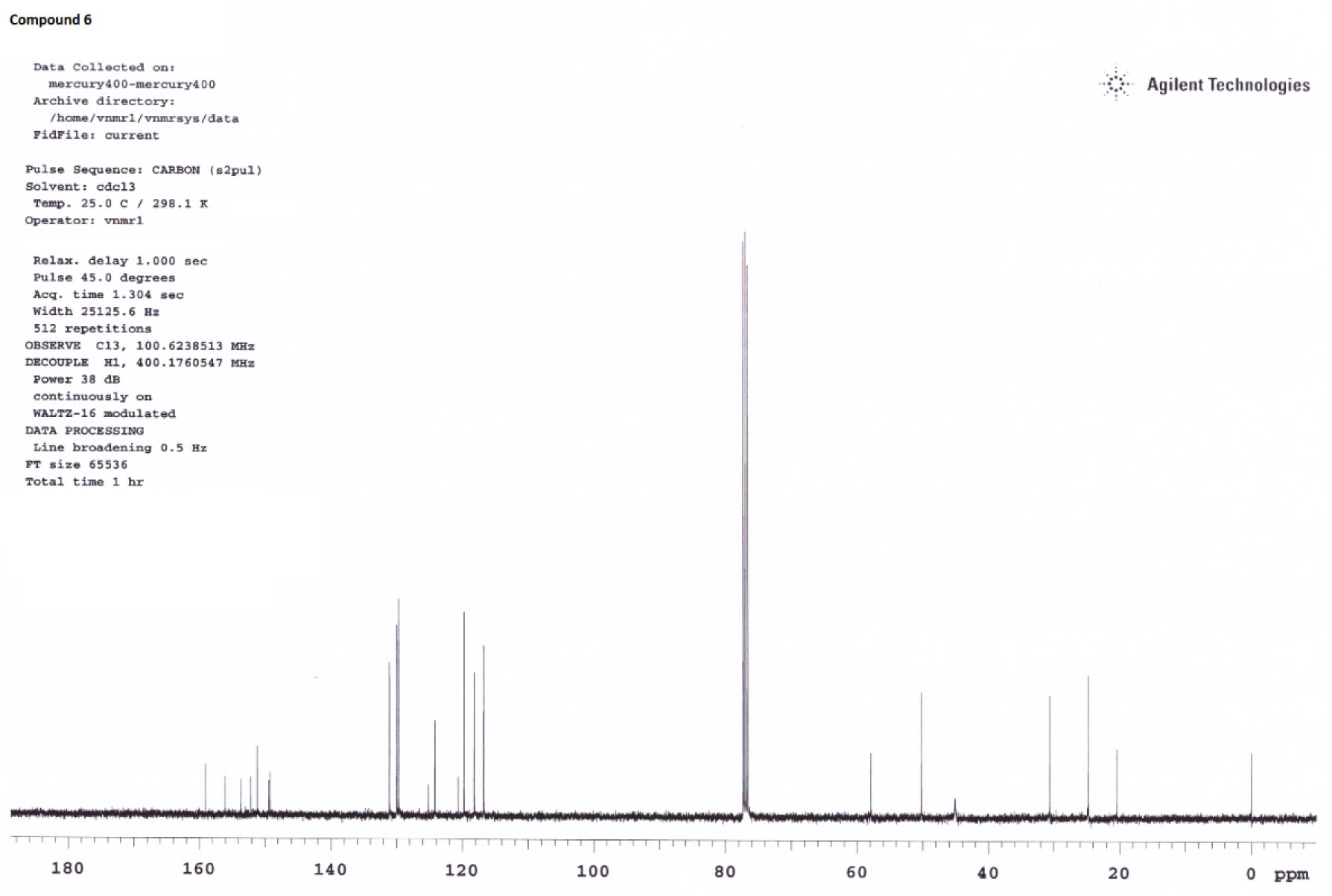

Supplement: Figure S8 — 13C NMR spectrum of Compound 6. [file tjc-48-01-0108s8.tif]

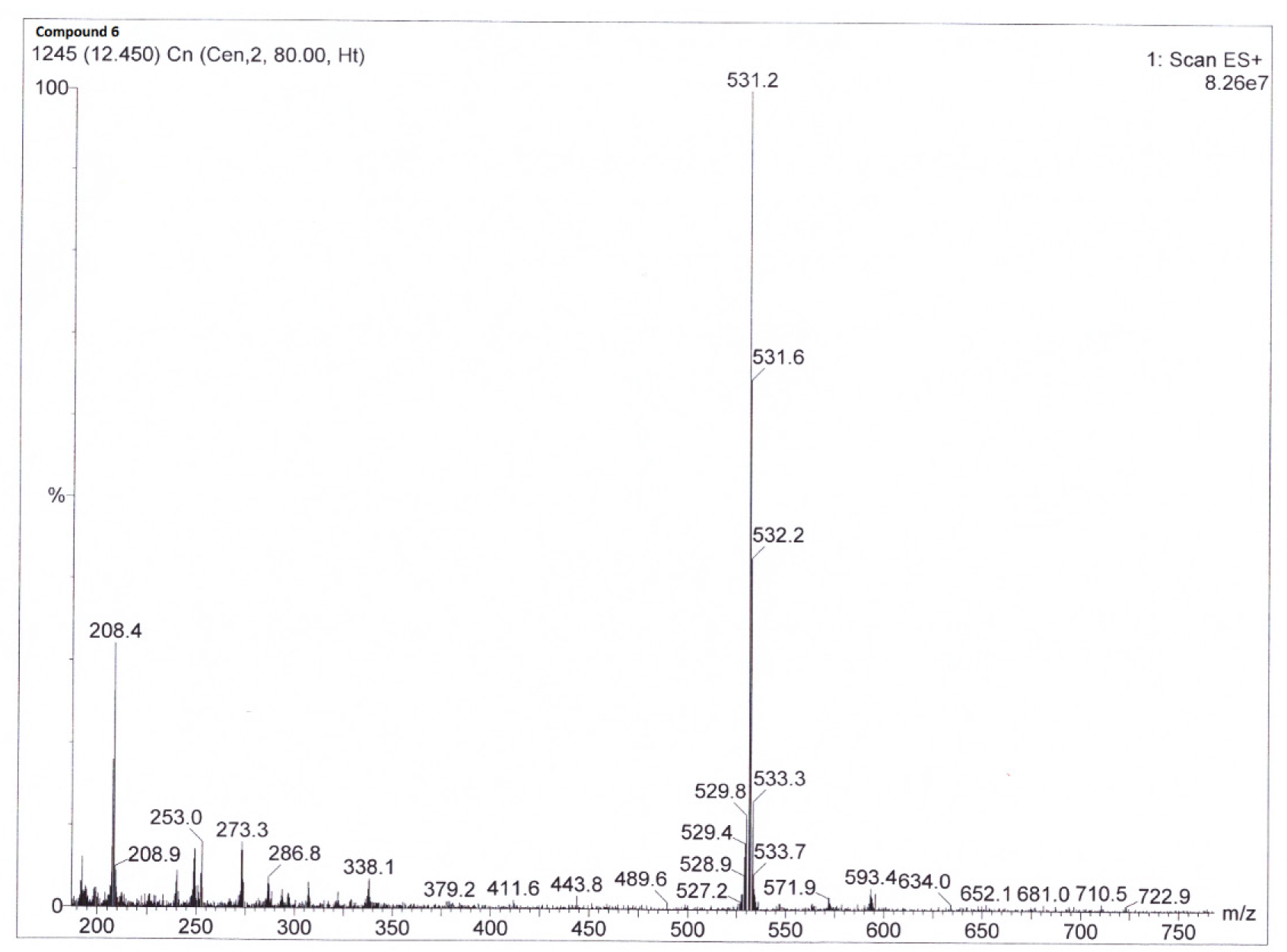

Supplement: Figure S9 — Mass spectrum of Compound 6. [file tjc-48-01-0108s9.tif]

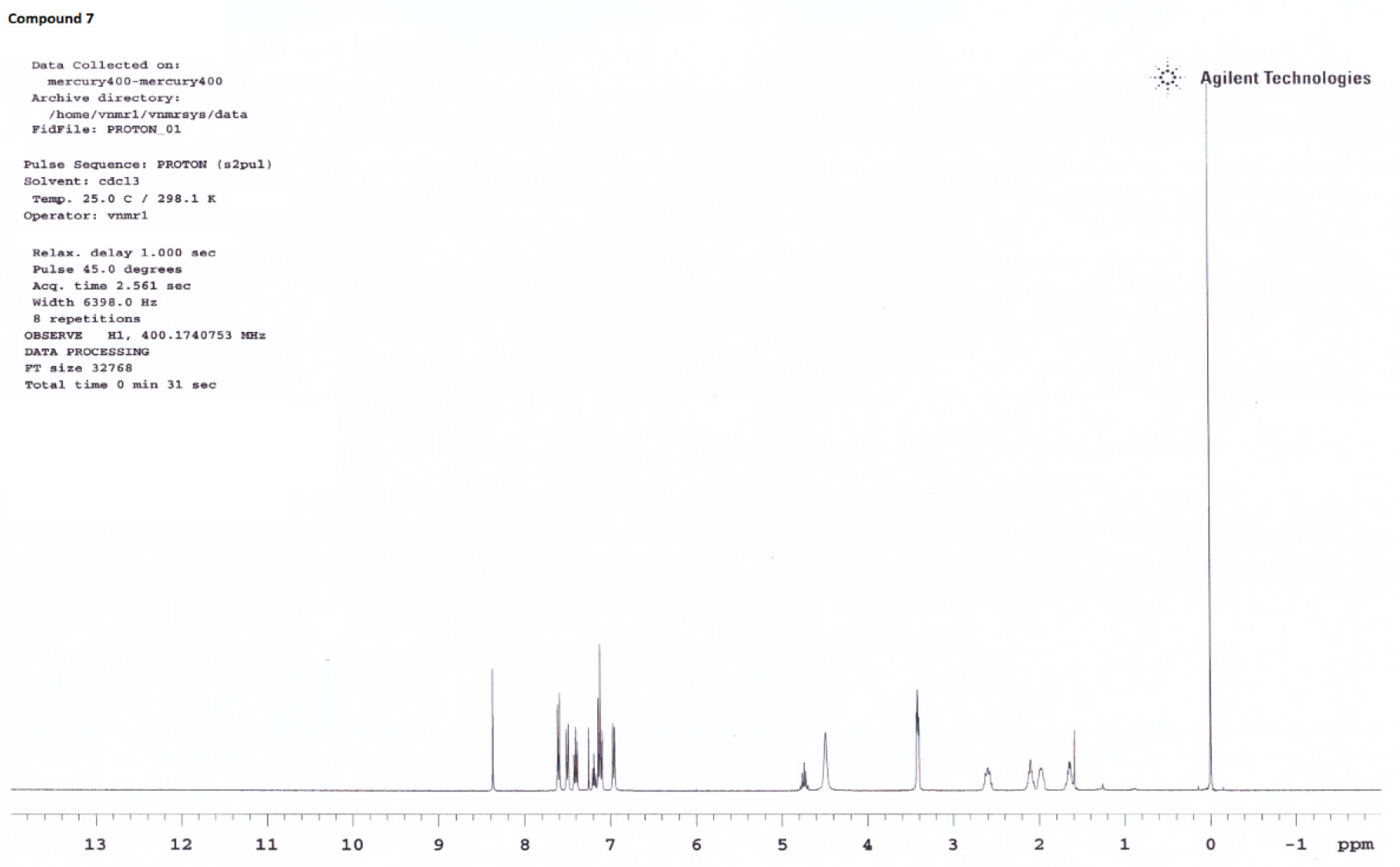

Supplement: Figure S10 — 1H NMR spectrum of Compound 7. [file tjc-48-01-0108s10.tif]

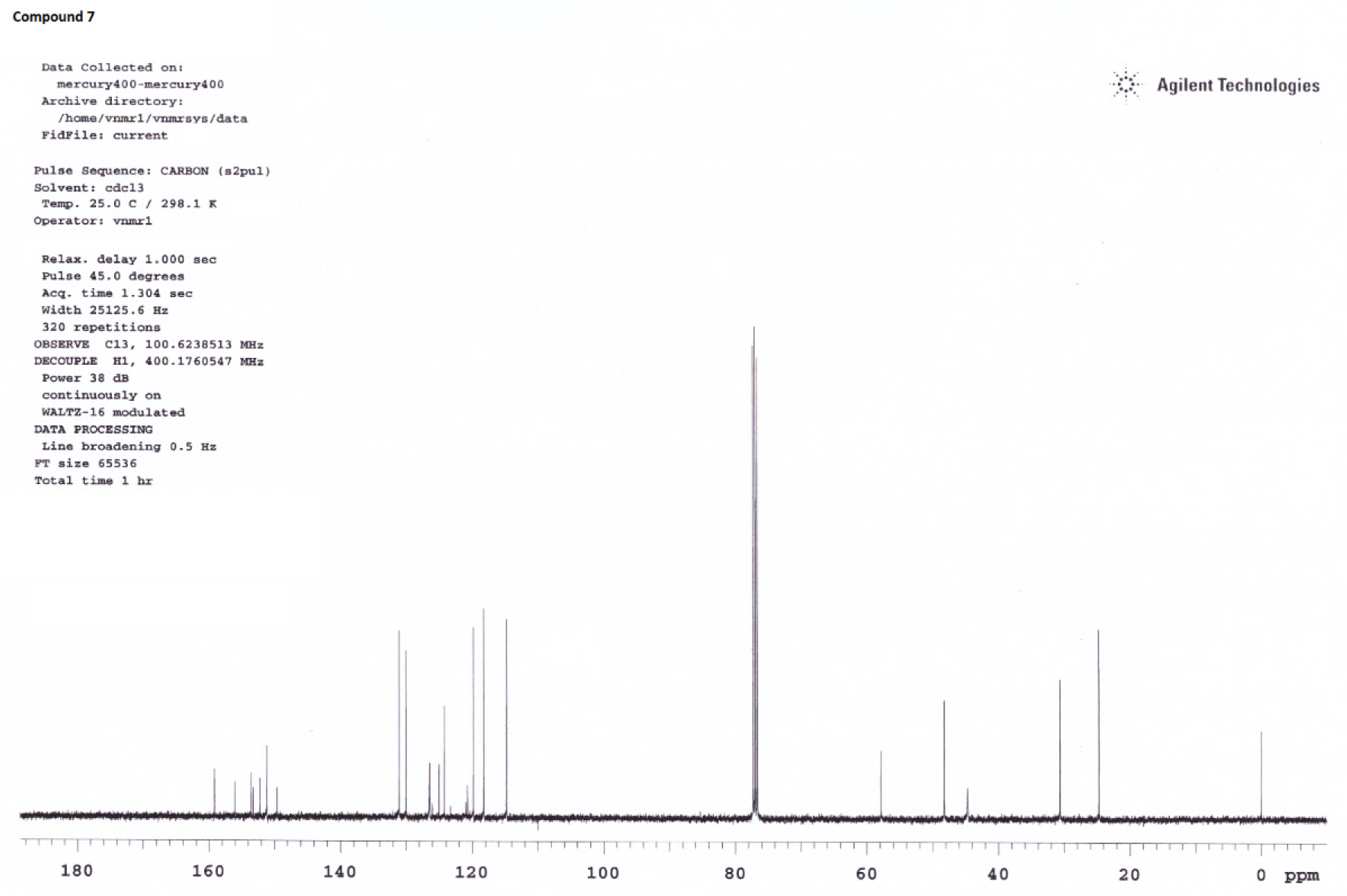

Supplement: Figure S11 — 13C NMR spectrum of Compound 7. [file tjc-48-01-0108s11.tif]

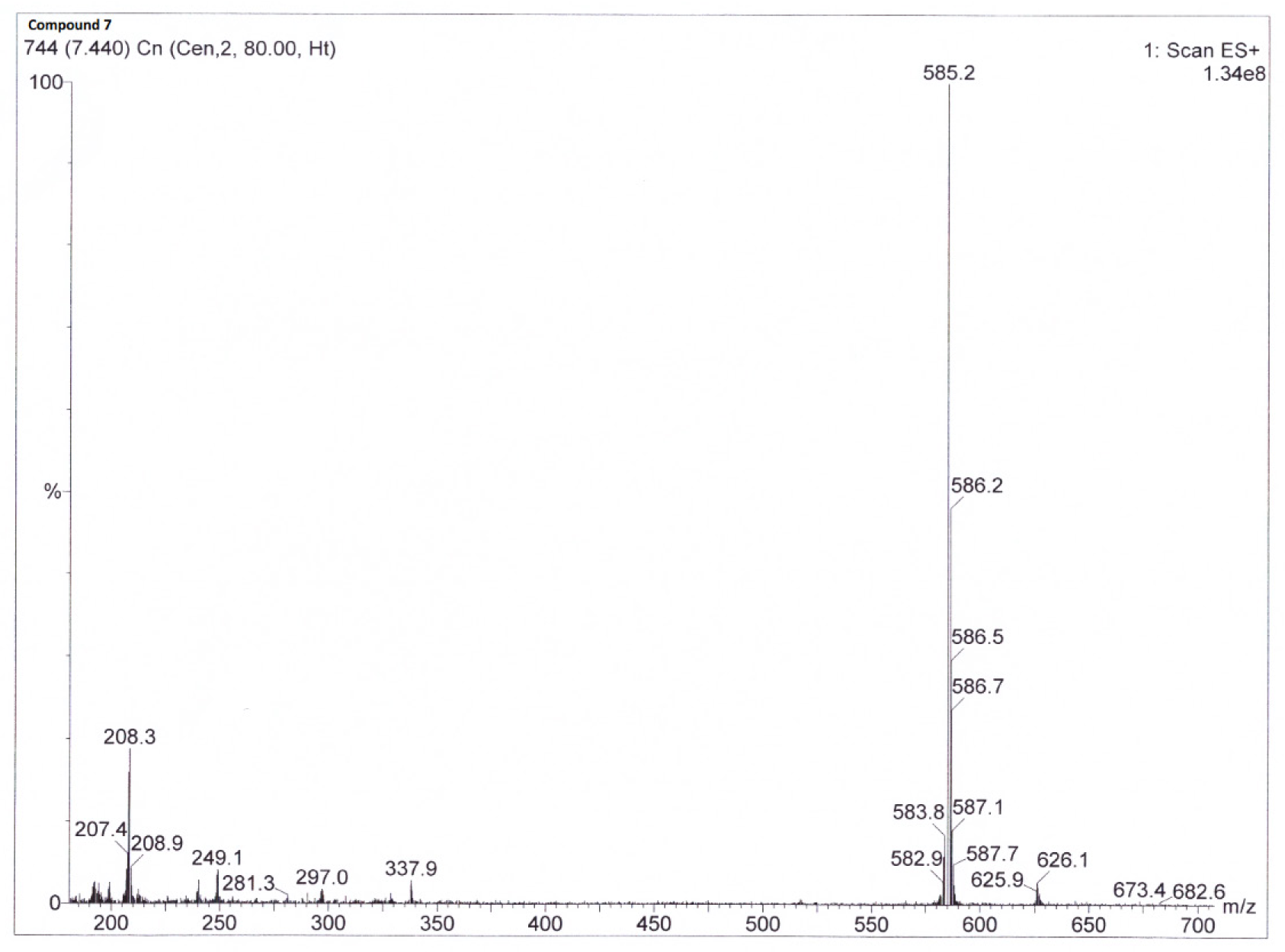

Supplement: Figure S12 — Mass spectrum of Compound 7. [file tjc-48-01-0108s12.tif]

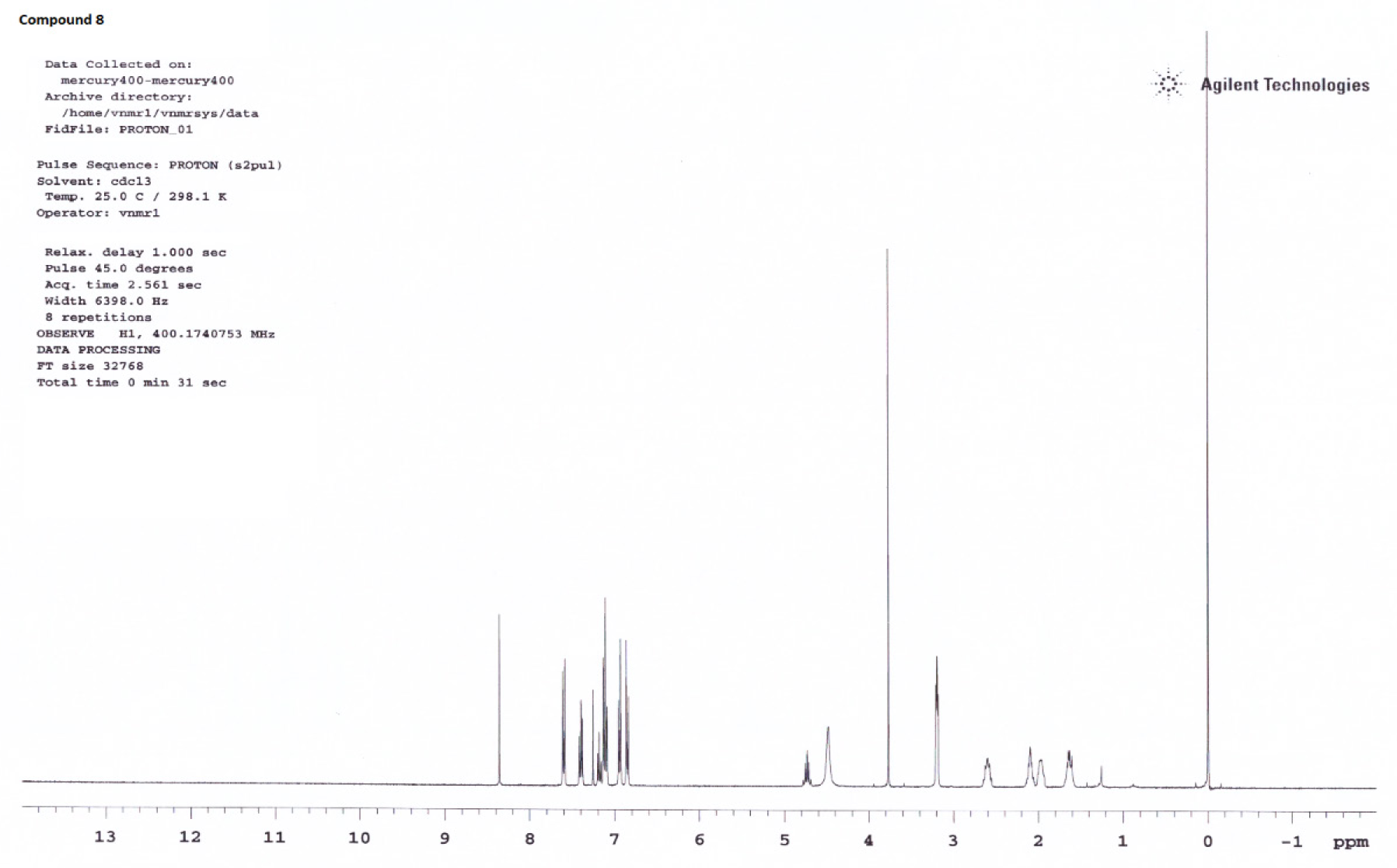

Supplement: Figure S13 — 1H NMR spectrum of Compound 8. [file tjc-48-01-0108s13.tif]

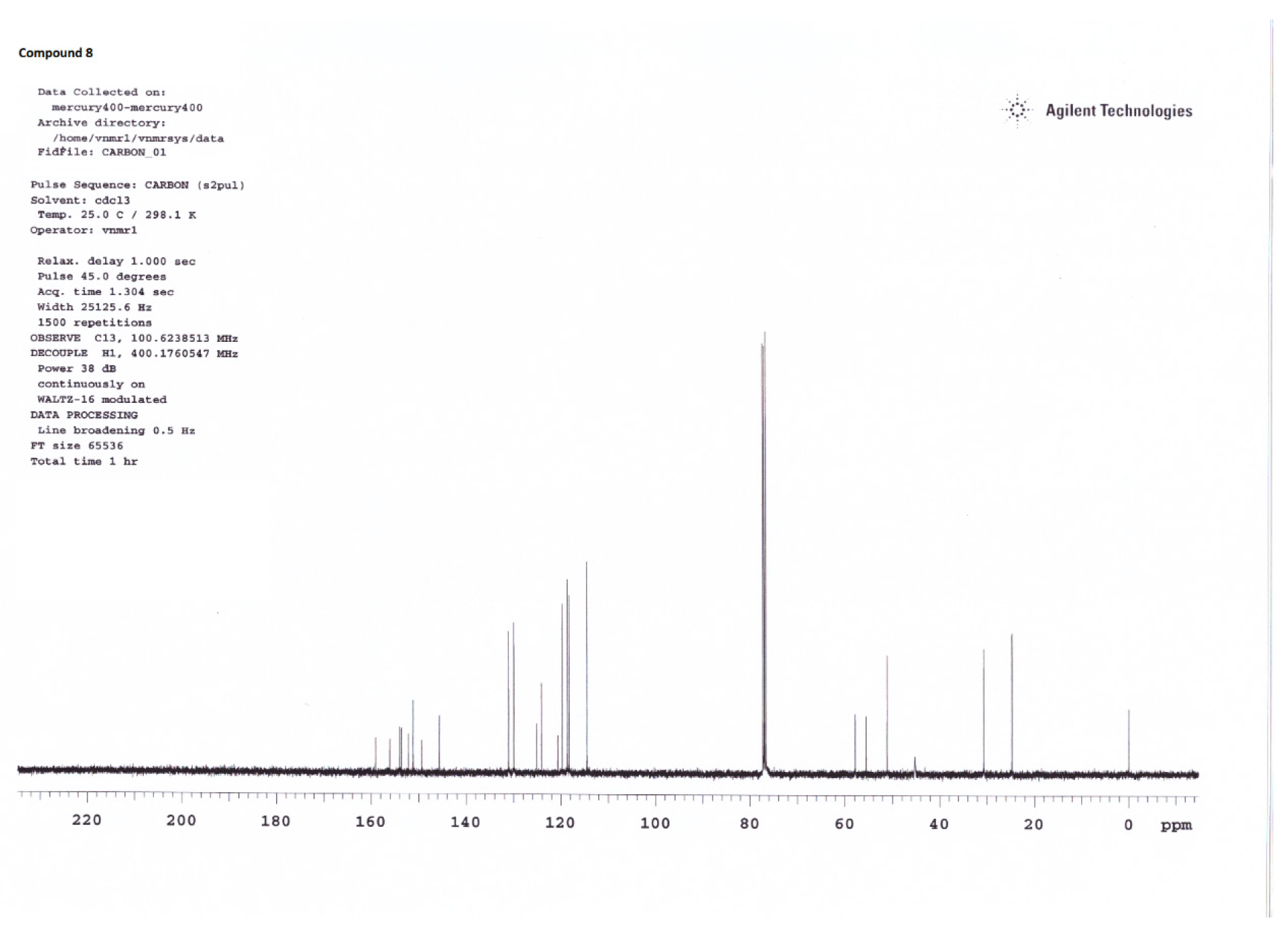

Supplement: Figure S14 — 13C NMR spectrum of Compound 8. [file tjc-48-01-0108s14.tif]

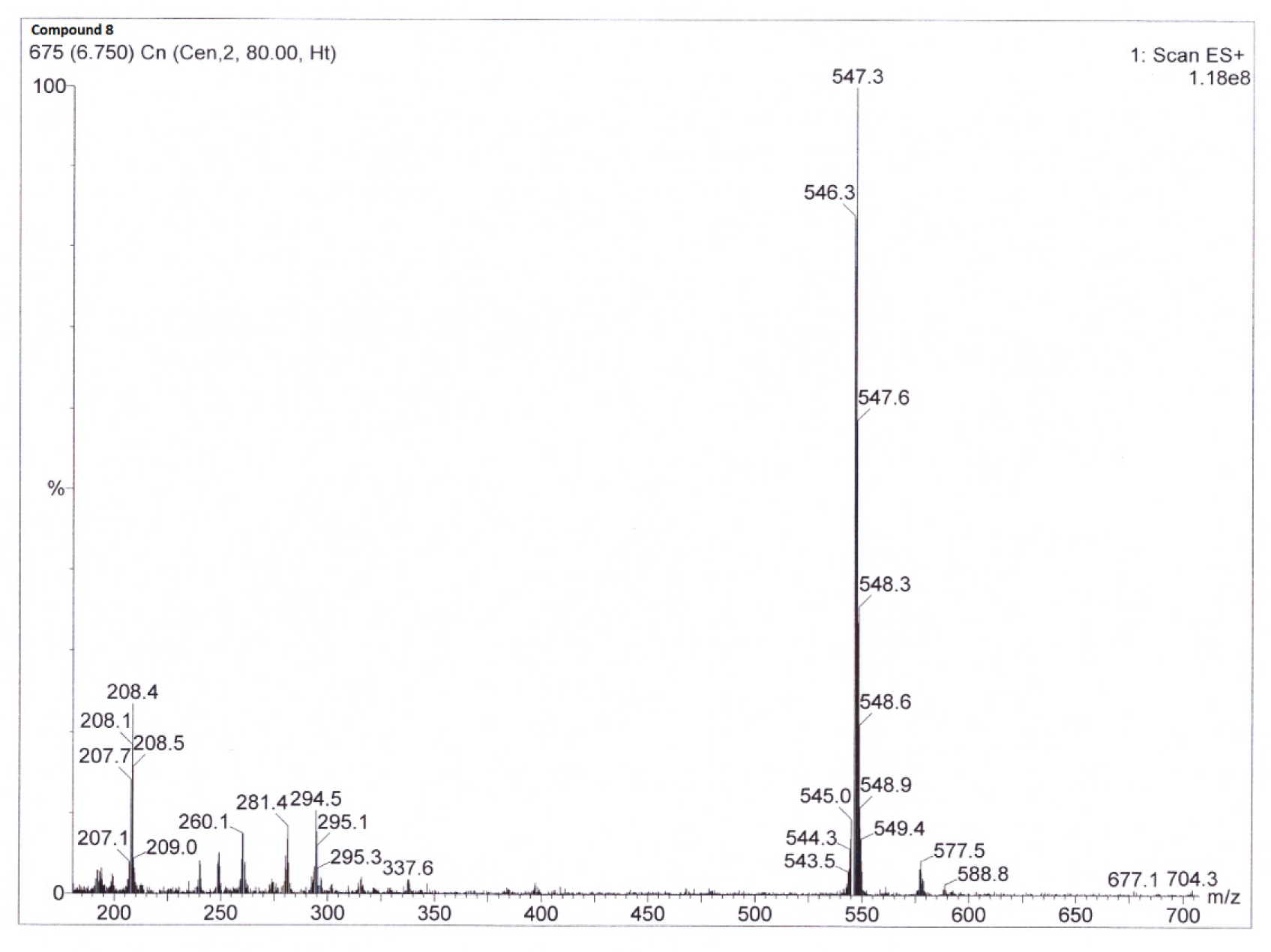

Supplement: Figure S15 — Mass spectrum of Compound 8. [file tjc-48-01-0108s15.tif]

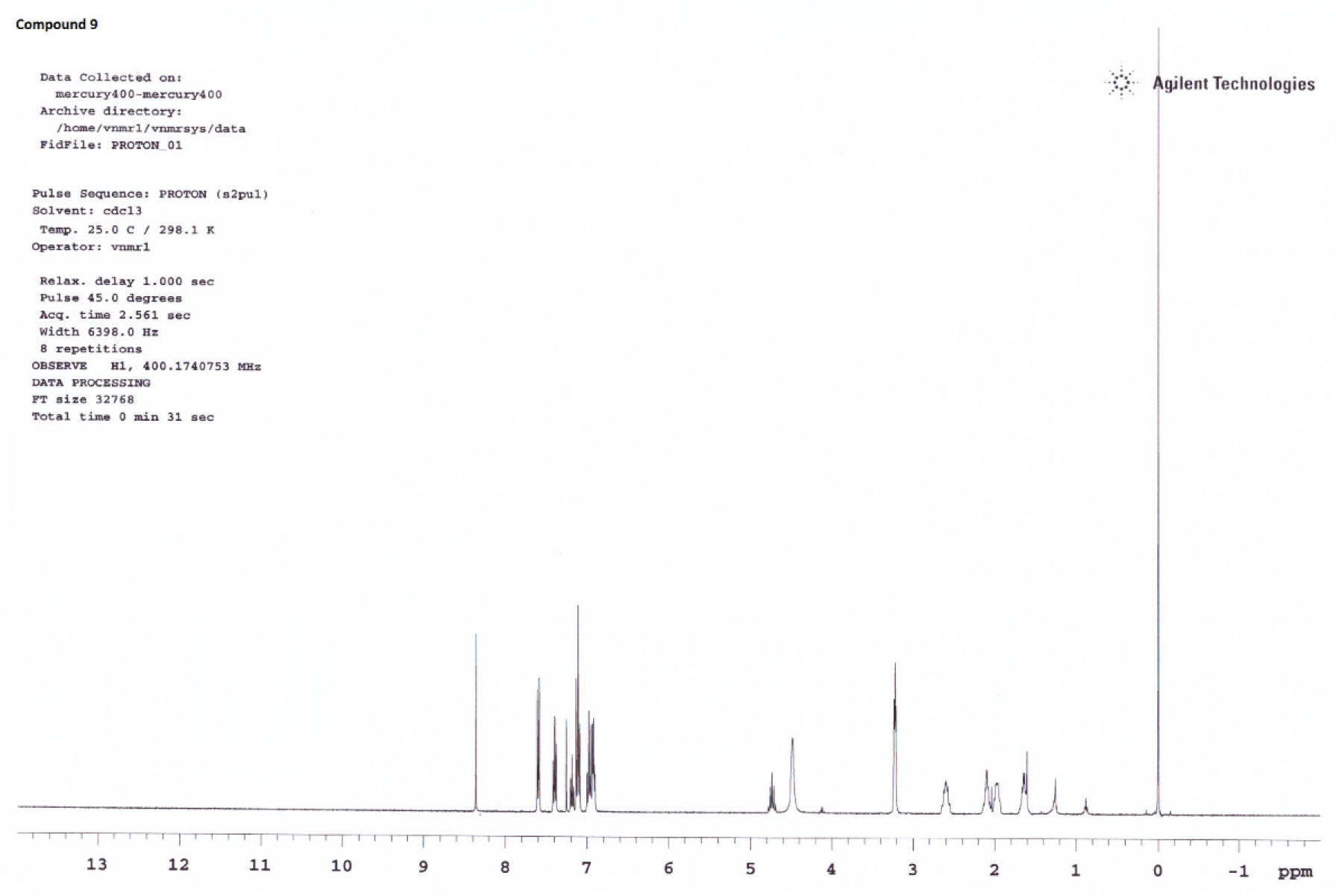

Supplement: Figure S16 — 1H NMR spectrum of Compound 9. [file tjc-48-01-0108s16.tif]

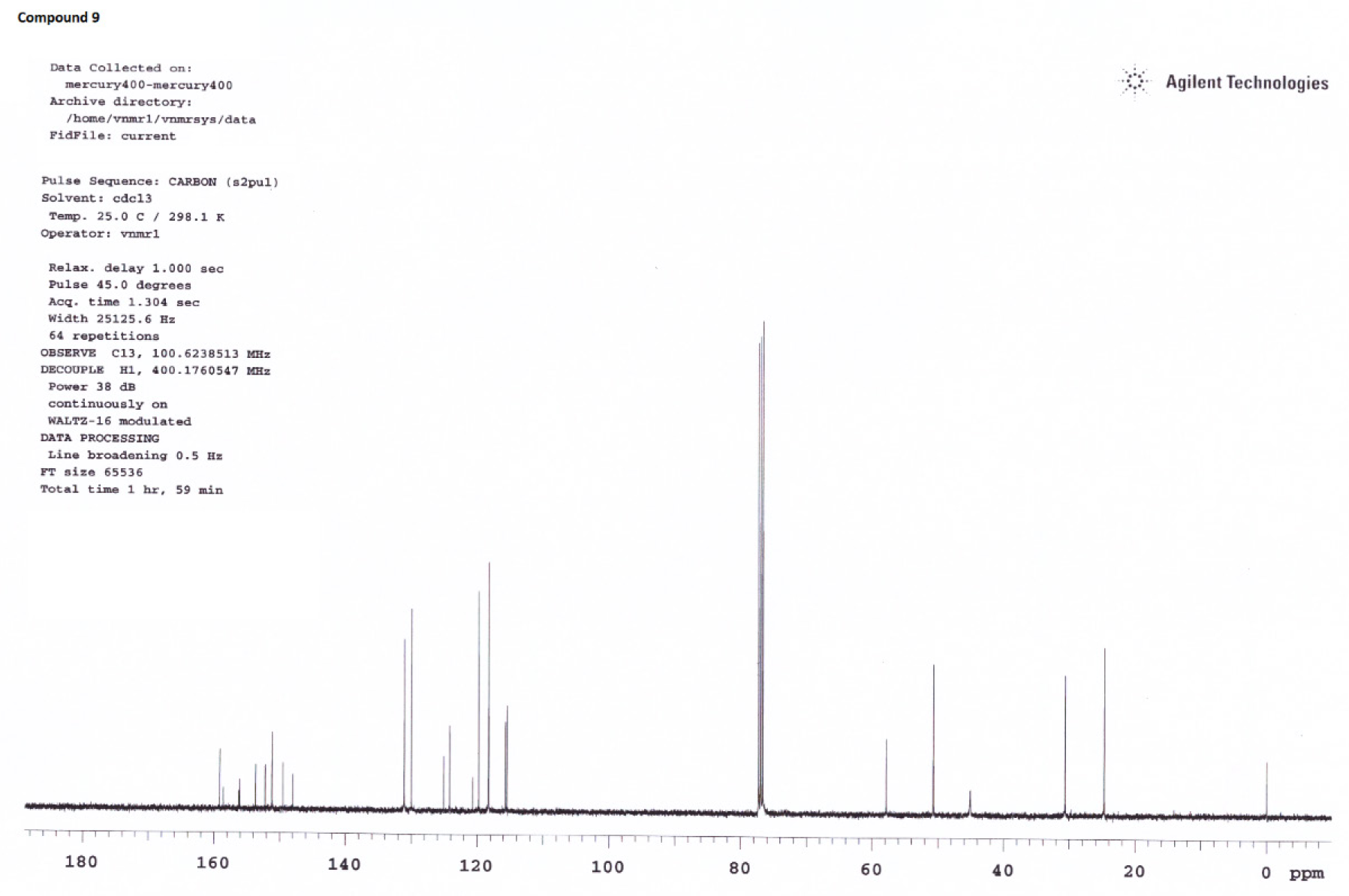

Supplement: Figure S17 — 13C NMR spectrum of Compound 9. [file tjc-48-01-0108s17.tif]

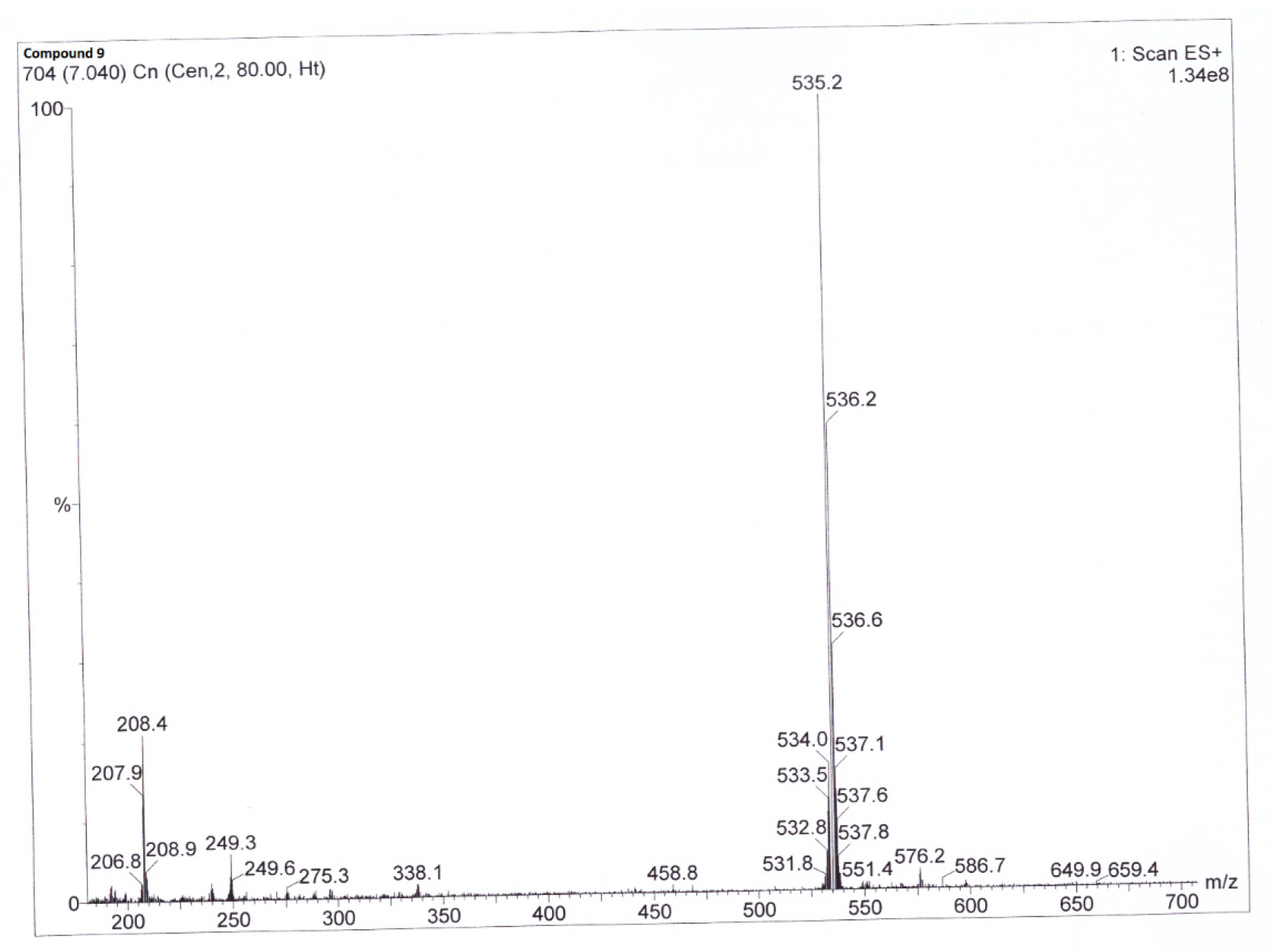

Supplement: Figure S18 — Mass spectrum of Compound 9. [file tjc-48-01-0108s18.tif]

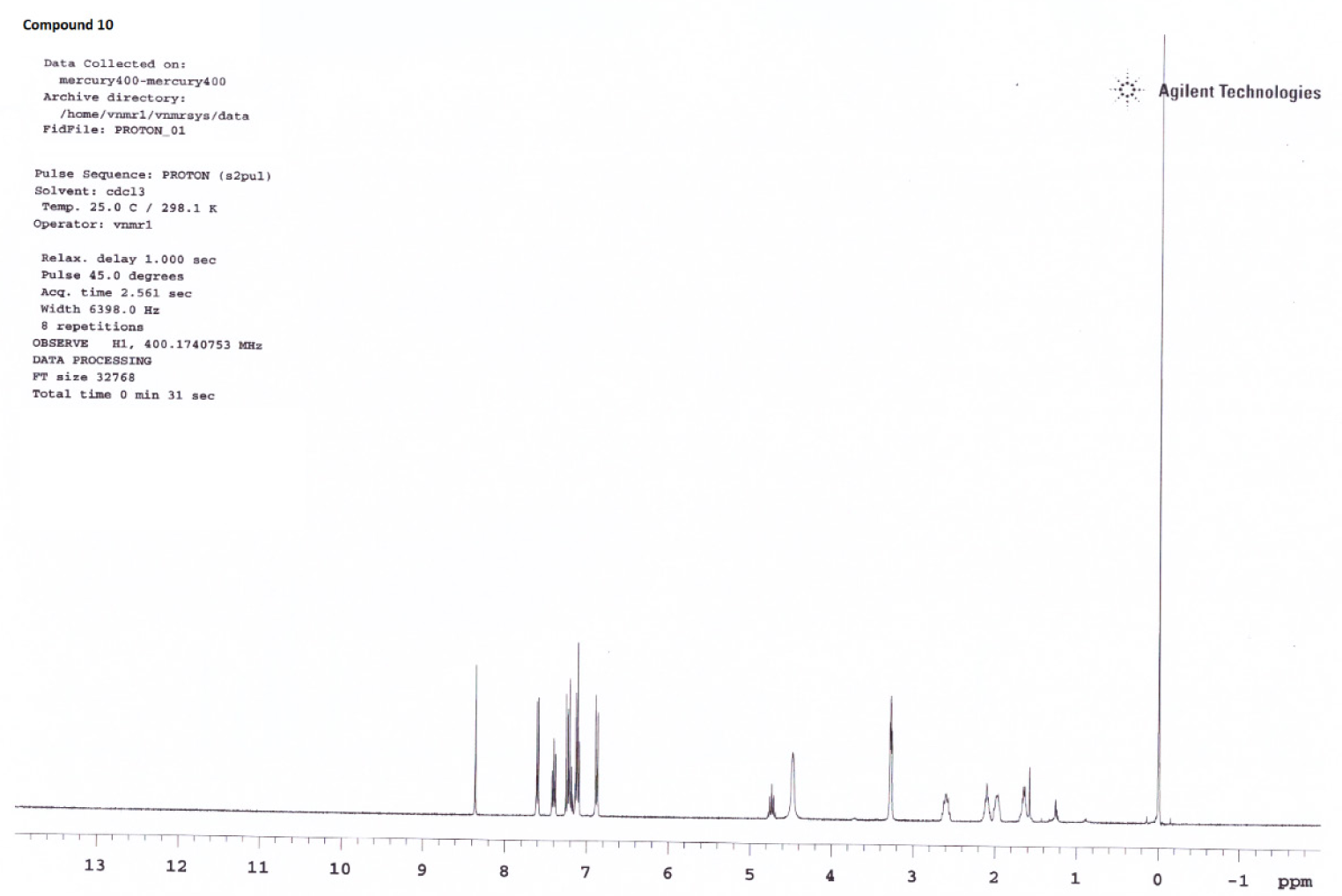

Supplement: Figure S19 — 1H NMR spectrum of Compound 10. [file tjc-48-01-0108s19.tif]

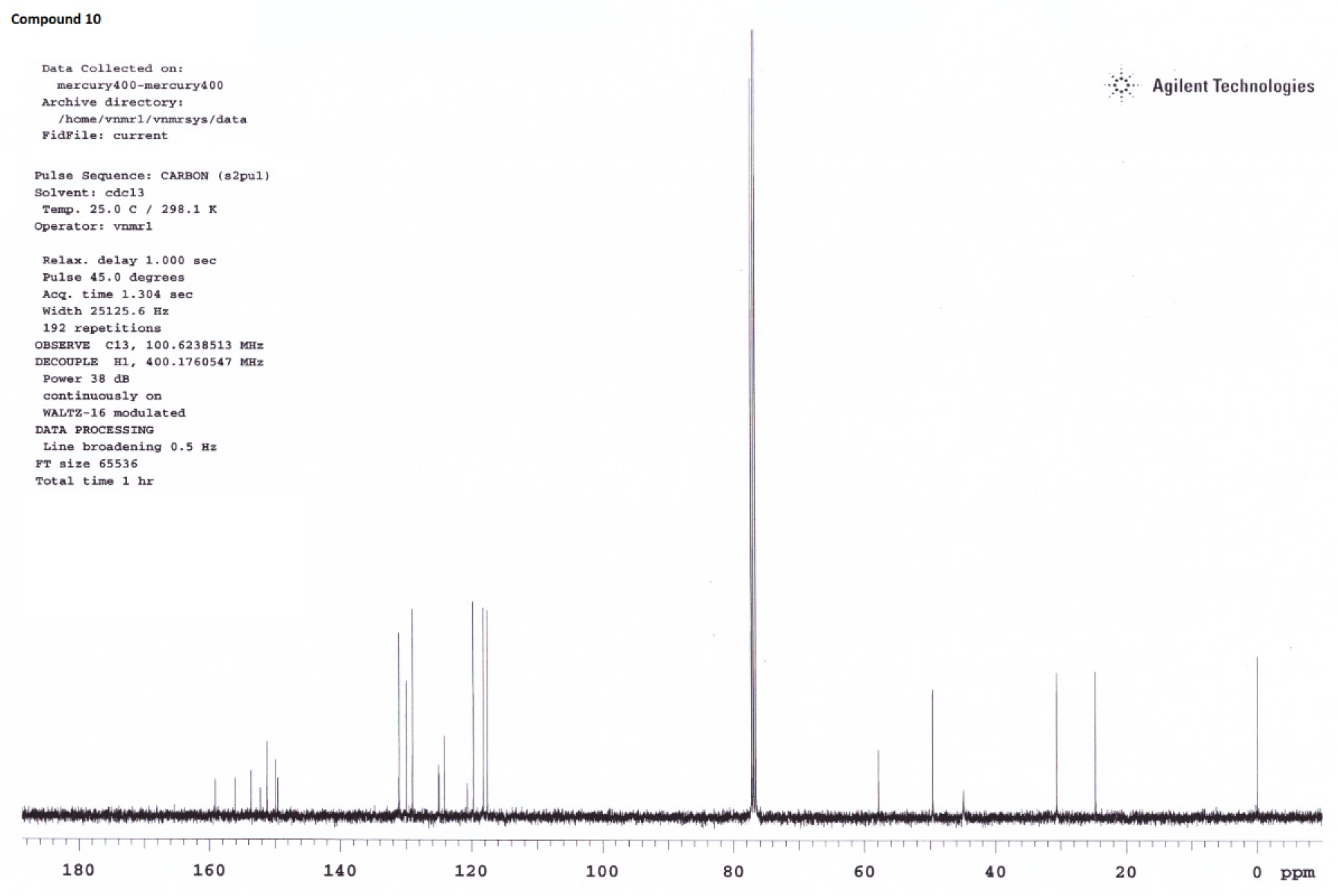

Supplement: Figure S20 — 13C NMR spectrum of Compound 10. [file tjc-48-01-0108s20.tif]

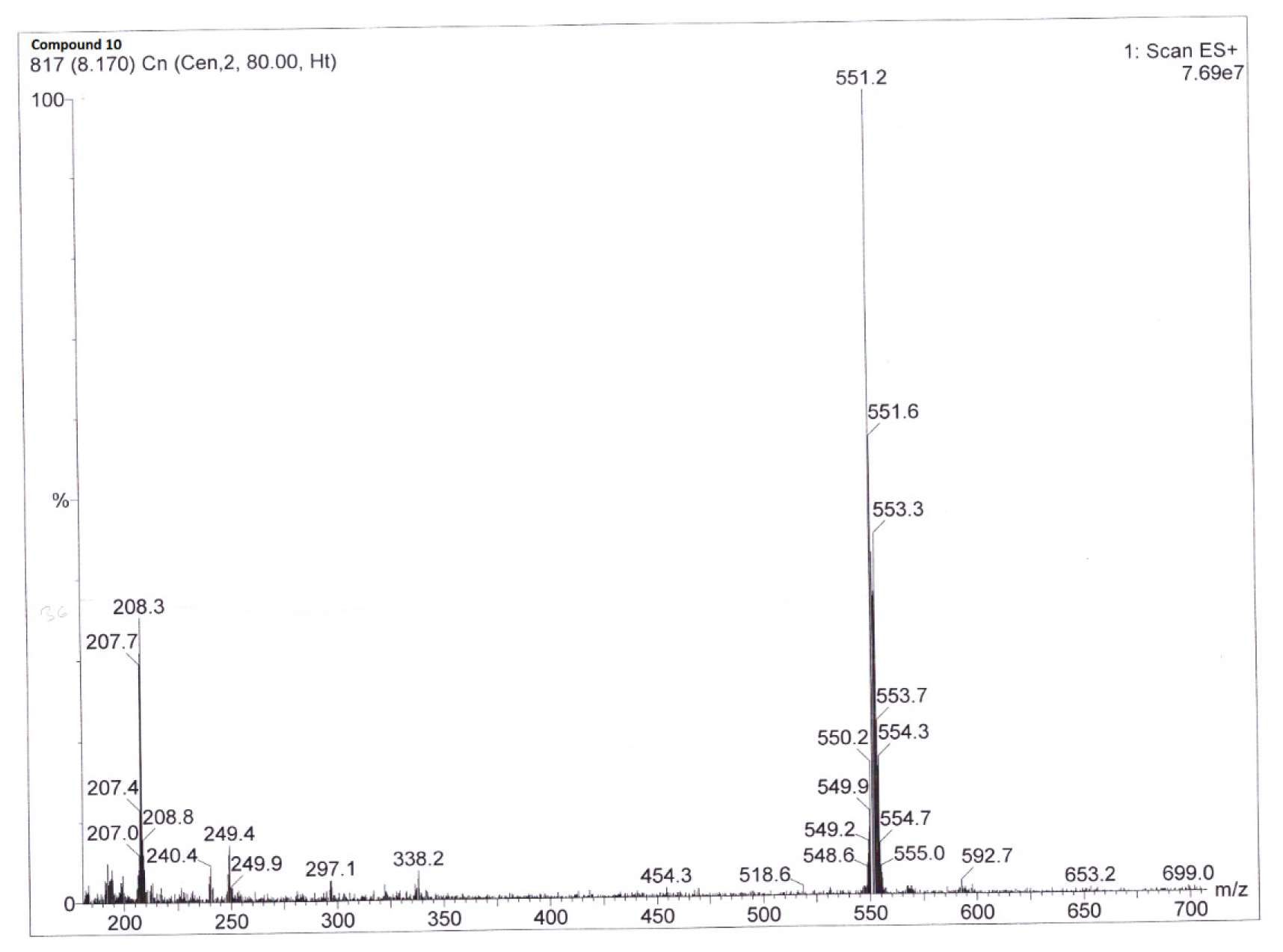

Supplement: Figure S21 — Mass spectrum of Compound 10. [file tjc-48-01-0108s21.tif]

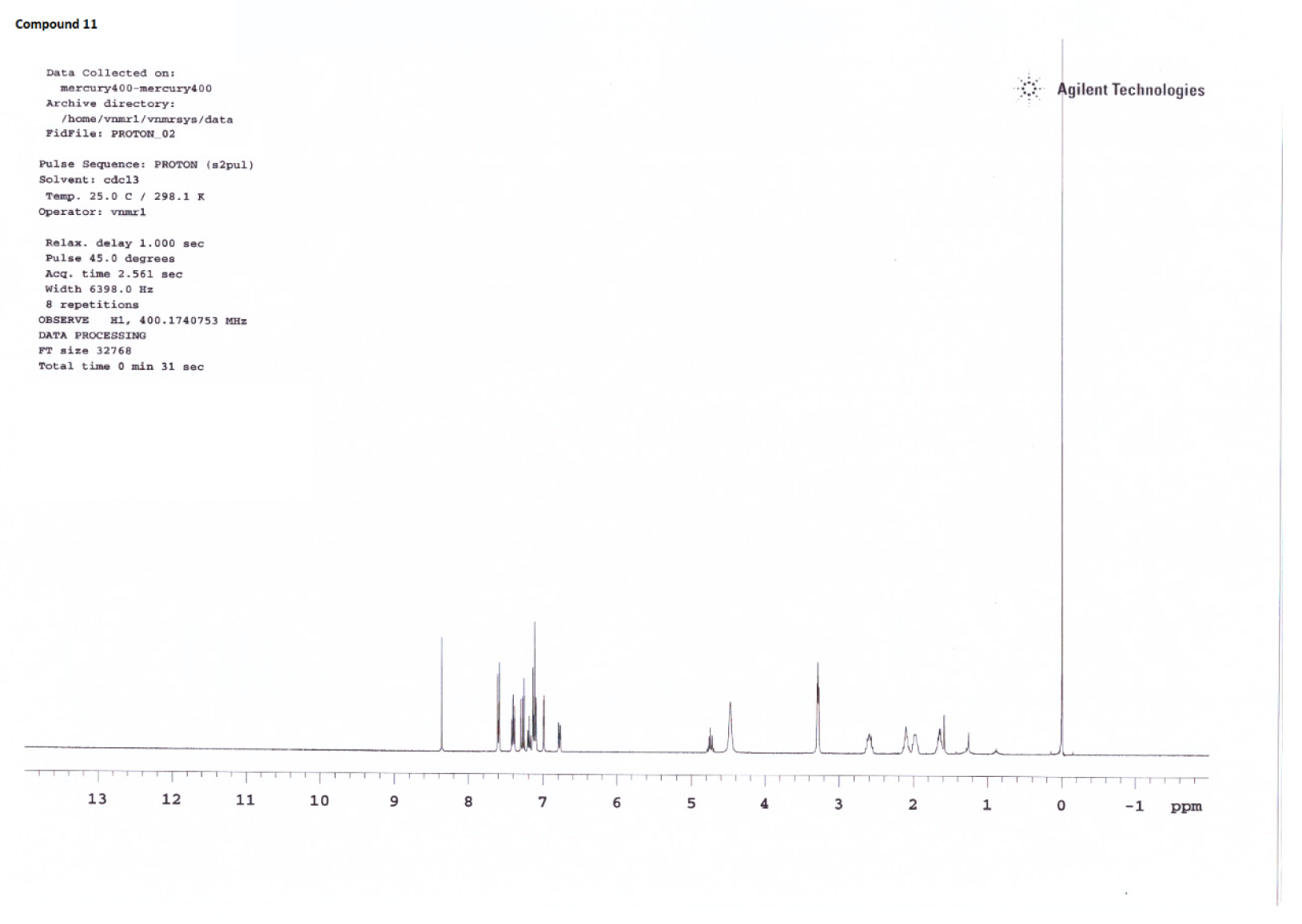

Supplement: Figure S22 — 1H NMR spectrum of Compound 11. [file tjc-48-01-0108s22.tif]

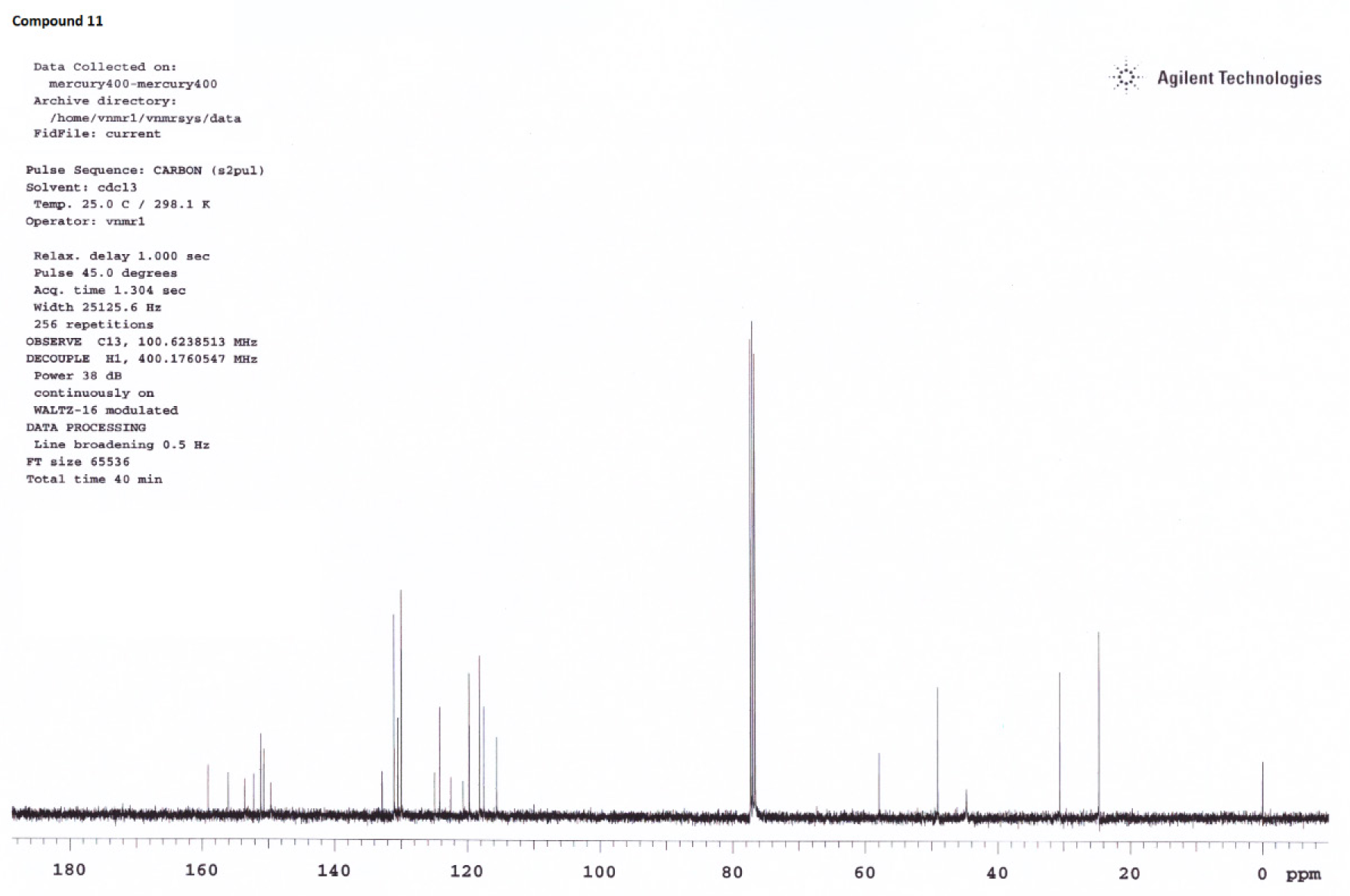

Supplement: Figure S23 — 13C NMR spectrum of Compound 11. [file tjc-48-01-0108s23.tif]

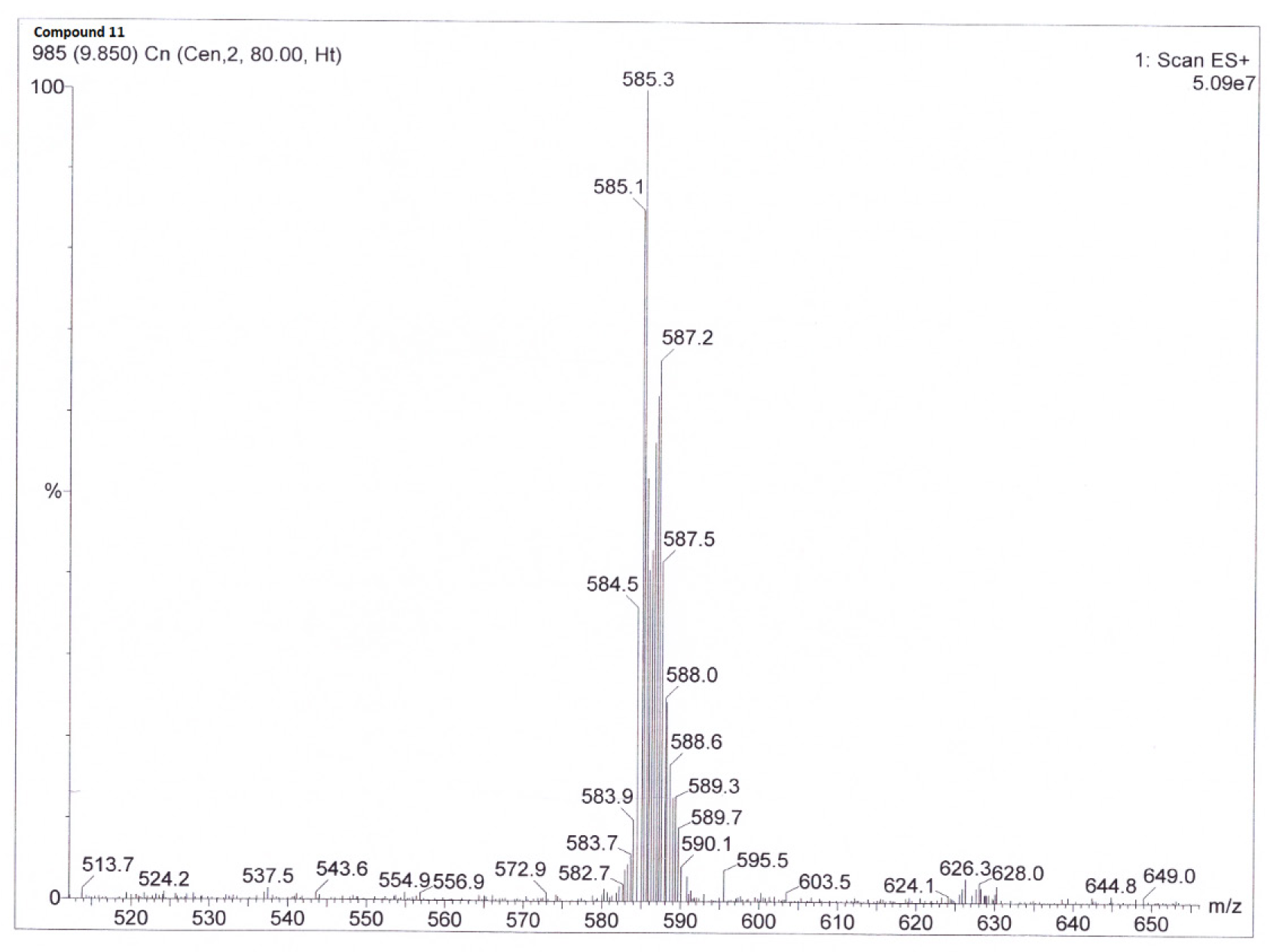

Supplement: Figure S24 — Mass spectrum of Compound 11. [file tjc-48-01-0108s24.tif]
